# A cohesin cancer mutation reveals a role for the hinge domain in genome organization and gene expression

**DOI:** 10.1371/journal.pgen.1009435

**Published:** 2021-03-24

**Authors:** Zachary M. Carico, Holden C. Stefan, Megan Justice, Askar Yimit, Jill M. Dowen

**Affiliations:** 1 Cancer Epigenetics Training Program, University of North Carolina at Chapel Hill, Chapel Hill, North Carolina, United States of America; 2 Integrative Program for Biological and Genome Sciences, University of North Carolina at Chapel Hill, Chapel Hill, North Carolina, United States of America; 3 Curriculum in Genetics and Molecular Biology, University of North Carolina at Chapel Hill, Chapel Hill, North Carolina, United States of America; 4 Department of Biochemistry and Biophysics, University of North Carolina at Chapel Hill, Chapel Hill, North Carolina, United States of America; 5 Department of Biology, University of North Carolina at Chapel Hill, Chapel Hill, North Carolina, United States of America; 6 Lineberger Comprehensive Cancer Center, University of North Carolina at Chapel Hill, Chapel Hill, North Carolina, United States of America; King’s College London, UNITED KINGDOM

## Abstract

The cohesin complex spatially organizes interphase chromatin by bringing distal genomic loci into close physical proximity, looping out the intervening DNA. Mutation of cohesin complex subunits is observed in cancer and developmental disorders, but the mechanisms through which these mutations may contribute to disease remain poorly understood. Here, we investigate a recurrent missense mutation to the hinge domain of the cohesin subunit SMC1A, observed in acute myeloid leukemia. Engineering this mutation into murine embryonic stem cells caused widespread changes in gene expression, including dysregulation of the pluripotency gene expression program. This mutation reduced cohesin levels at promoters and enhancers, decreased DNA loops and interactions across short genomic distances, and weakened insulation at CTCF-mediated DNA loops. These findings provide insight into how altered cohesin function contributes to disease and identify a requirement for the cohesin hinge domain in three-dimensional chromatin structure.

## Author summary

Mammalian genomes consist of multiple meters of DNA which must be highly folded in order to fit inside of the nucleus. This folding is regulated at multiple scales by different biological mechanisms. The spatial organization of the genome is closely linked to its function, including the spatial and temporal expression of genes. Especially important for gene control is the partitioning of chromosomes into DNA loops, which are formed when two distal loci are brought into close contact. The folding of the genome into DNA loops is performed by cohesin and CTCF. The molecular basis for how DNA loops dynamically form and function in gene control is poorly understood. Here, we investigate a recurrent cancer mutation in cohesin and show that it causes altered folding of the genome into DNA loops and misexpression of many genes. This finding is important because cohesin mutations are common in many cancers and yet there is little understanding of how cohesin defects may contribute to disease.

## Introduction

Three-dimensional genome organization is an important regulator of gene expression in metazoans [1]. DNA loops form when two distal genomic sites are brought into physical contact, causing the intervening DNA to form a loop. Two major classes of DNA loops have been identified: E-P loops are formed when enhancers are brought in proximity to promoters and are associated with active gene expression, while insulated loops are formed by the interaction of a pair of distal CTCF-bound sites. Insulated loops can restrict the formation of potential E-P loops and act as a boundary element separating distinct chromatin states. E-P loops are developmentally dynamic and associated with active transcription, while insulated loops may be shared across cell types and independent of the transcriptional state contained within them [2–5]. The impact of aberrant DNA looping on gene expression in human disease contexts remains unclear.

Formation of DNA loops is mediated by the cohesin complex, which is comprised of a core structure consisting of the proteins SMC1A, SMC3, and RAD21 [6]. SMC1A and SMC3 each have large flexible coiled-coil domains, a hinge domain and an ATPase domain. In the complex, SMC1A and SMC3 heterodimerize via interaction between their coiled-coil domains, hinge domains and ATPase domains [7,8]. The ATPase domains are themselves bound by the kleisin subunit RAD21 [9–11]. Several additional proteins from the HEAT-repeat-containing family, such as STAG1, STAG2, PDS5A, PDS5B, NIPBL, and WAPL, associate with the core complex through interactions with RAD21 [12]. Both the core cohesin ring and HEAT-repeat proteins may directly interact with DNA [10,13,14]. In addition to its roles in regulating DNA loop formation, cohesin is required for sister chromatid cohesion and DNA damage repair mechanisms in eukaryotes [15]. How the cohesin complex forms loops and maintains normal cellular function are major open questions.

Altered cohesin function is observed in human disease [16]. Germline mutations in several cohesin subunits cause a range of developmental defects that are collectively termed cohesinopathies [17]. Somatic mutations in cohesin subunits are implicated in a wide array of cancers [18–20]. The *SMC3* and *RAD21* genes are haploinsufficient, as demonstrated by heterozygous frameshift and nonsense somatic mutations [20]. The *STAG2* gene is encoded on the X chromosome and its inactivation is only partially compensated for by its paralogue, *STAG1* [20]. Reduced expression of these subunits results in pre-leukemic skewing of hematopoiesis and contributes to myeloid neoplasia in murine models [21–24]. The X chromosome-encoded *SMC1A* gene is subject to missense mutations, which are distributed along the length of the coding sequence but enriched at several hotspots [25]. While these hotspots often occur at highly conserved residues within the characterized ATPase and hinge domains [25], it is not known whether they impact cohesin function. Mechanistic insight into how cohesin mutations contribute to malignancy is lacking. Previous studies have largely ruled out models where cohesin defects cause catastrophic chromosome damage via aneuploidy, chromosomal breaks, and impaired sister chromatid cohesion [21,26]. In an alternative model, aberrant cohesin function caused by mutation to cohesin subunits, may lead to disease by misregulating chromatin loops and gene expression [27]. Accordingly, mutations that cause partial loss of function may reveal important mechanistic insights into cohesin function.

Understanding how aberrant cohesin function causes human disease is complicated by limited knowledge of the molecular mechanisms through which cohesin interacts with chromatin and mediates DNA looping. Studies in yeast have demonstrated that ATP hydrolysis by cohesin is required for loading onto chromatin, translocation, and establishment of sister chromatid cohesion, while mammalian studies found that ATP hydrolysis is required for loop extrusion [28–31]. Cohesin ATPase activity in both yeast and metazoans is stimulated by Scc2/NIPBL, while evidence in yeast suggest that ATPase activity and NIPBL incorporation into the complex may be inhibited by Pds5 [29,32]. An intact SMC1A-SMC3 hinge is also required for cohesin function. In yeast, mutation of residues that stabilize the interface between the Smc1 and Smc3 hinge subunits decreases cohesin levels on chromatin, while neutralization of a positively-charged channel created by that interface permits loading but prevents establishment of sister chromatid cohesion [31,33,34]. Given that cohesin is thought to use the ATPase domains and kleisin subunit to capture DNA [35], it is important to understand the molecular details through which the hinge modulates cohesin function.

We hypothesized that recurrent cancer mutations to cohesin subunits might disrupt important functions and, therefore, provide mechanistic insight into cohesin biology and the role of cohesin in oncogenesis. We use publicly available cancer exome sequencing data to identify a hotspot mutation at amino acid residue 586 in the hinge of SMC1A. Engineering an arginine (R) to tryptophan (W) mutation at this position in the genome of murine embryonic stem cells (mESCs) caused drastic changes in gene expression, reduced cohesin binding to DNA and loss of chromatin loops. These findings represent the first functional assessment of the cohesin hinge in mammalian cells and provide molecular insights into aberrant cohesin function during disease states.

## Results

### A leukemia mutation causes global dysregulation of gene expression in mESCs

**Fig 1:**
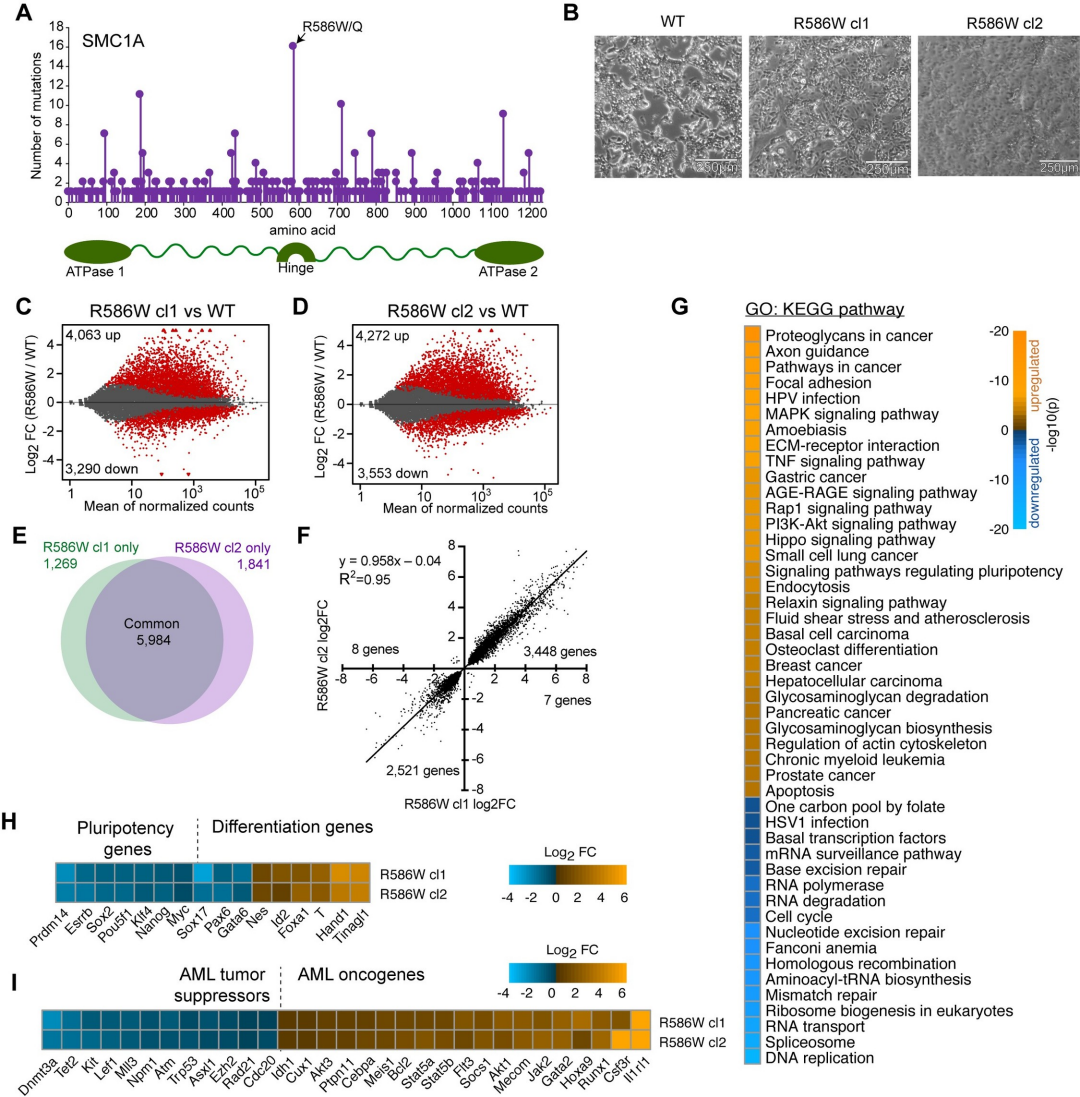
Broad transcriptional changes in SMC1A-R586W mESCs. **A** Lollipop plot of protein sequence mutations observed in SMC1A in the COSMIC cancer genome atlas. **B** Images of wildtype and two independently derived SMC1A-R586W ES clones growing in complete media with LIF. Scale bar, 500μm. **C-D** MA plots comparing RNA-seq data from R586W clone (cl) 1 and cl2 to wildtype mESCs. n = 3 biological replicates for all groups. Red, adjusted p<0.1 as determined by DESeq2. **E** Venn diagram showing overlap of differentially-expressed (DE) genes between two R586W ES clones. **F** Correlation plot of 5,984 common differentially expressed genes in R586W cl1 and R586W cl2. **G** Gene ontology analysis using upregulated and downregulated DE genes shared by R586W cl1 and cl2. **H** Expression of pluripotency genes and **I** AML tumor suppressors and oncogenes in R586W cl1 and cl2.

Using the COSMIC cancer-genome database we identified a recurrent R-to-W mutation at SMC1A amino acid residue 586 that occurred almost exclusively in the context of acute myeloid leukemia (AML)(**Fig 1A**) [25]. R586 is thus far the single most frequently mutated SMC1A residue in AML (**Fig 1A**). Roughly half (7 out of 16) of the mutations at R586 resulted in a substitution to tryptophan (W), while the rest (9 out of 16) were changed to glutamine (Q). As revealed by crystallography and cryo-EM structures, R586 is on a solvent-exposed outer surface of the cohesin hinge, making it a candidate site for post-translational modifications or mediating interactions with other proteins or nucleotides (**S1A Fig**) [13,34]. Importantly, the cohesin hinge interacts with single stranded DNA and STAG proteins, catalyzes ATP hydrolysis by the head domains, and is thought to be important for dynamic changes in cohesin conformation and movement (**S1A Fig**) [13,36]. R586 is not found at the SMC1A-SMC3 hinge interface, like other mutations which have been shown to destabilize cohesin ring closure in yeast [31,34]. Furthermore, SMC1A-R586 is conserved in metazoans but not yeast, which do not generally utilize DNA loops to control gene expression (**S1B Fig**). Given the position of the residue within a potentially functional region of SMC1A, and its prevalence in disease, we sought to evaluate whether this mutation alters cohesin function.

To study SMC1A-R586, we used CRISPR/Cas9 genome editing to generate two independent murine embryonic stem cell (mESC) clonal lines bearing the R586W mutation encoded in the endogenous *Smc1a* locus. Since *Smc1a* is encoded on the X chromosome and the mESC V6.5 line is male, the cells express mutant SMC1A protein and no wildtype protein. mESCs provide an isogenic background in which to study direct effects of a single mutation, independent of other potentially confounding mutations in a cancer context. Additionally, embryonic stem cells represent a powerful system for the study of transcriptional regulation and cell identity due to the wealth of previous information and their ability to both self-renew and give rise to all cell types of an adult organism. Since a large number of cancers, and especially blood cancers, exhibit loss of cellular identity [37], knocking the SMC1A-R586W mutation into mESCs allows for investigation of potential aberrant cellular identity and the molecular mechanisms that underlie it. The SMC1A-R586W mutation did not significantly alter expression levels of SMC1A or other cohesin complex subunits (**S1C and S1D Fig**). Morphologically, SMC1A-R586W mESCs adopt a flattened and differentiated appearance, similar to that previously reported for SMC1A knockdown (**Fig 1B)** [38].

We assessed whether gene expression is altered in SMC1A-R586W mESCs by performing RNA-seq on wildtype and each SMC1A-R586W clonal mESC line. Using an adjusted p-value of 0.1, over 7,000 differentially-expressed genes (DEGs) were identified in each SMC1A-R586W clone relative to wildtype, with somewhat more genes increased in expression than decreased (**Fig 1C and 1D and S1 Table**). The two mutant clones exhibited similar gene expression changes: Of the more than 7,000 DEGs in each SMC1A-R586W clone, nearly 6,000 are in common and display positively correlated changes relative to wildtype (**Fig 1E and 1F**). Principal component analysis revealed tight clustering by genotype and sequencing data were generally of good quality (**S1E Fig and S2 Table**). Gene ontology (GO) analysis showed that upregulated genes were enriched for signatures of cancer-associated gene expression programs, while downregulated genes were enriched for genes regulating transcription and chromatin state, DNA repair, and cell cycle (**Fig 1G and S3 Table**). Genes associated with pluripotency were reduced in expression, while differentiation-associated genes were increased (**Figs 1H and S1F**). Powerful enhancer elements termed Super-enhancers (SEs) are required to drive the expression of pluripotency-controlling transcription factors, while factors driving differentiation are encoded within DNA loops decorated by Polycomb machinery (Polycomb Domains; PDs) in mESCs [2,39,40]. Strikingly, DEGs controlled by SEs tended to exhibit substantial reductions in gene expression, while those repressed by Polycomb tended to undergo large increases (**S1G Fig**). Furthermore, the SMC1A-R586W mutation alters expression of several AML tumor suppressors and oncogenes in mESCs, including *Dnmt3a*, *Tet2*, *Ezh2*, and *Runx1*, suggesting a role for cohesin in regulating expression of these genes across cell types (**Fig 1I**) [41].

Next, we explored whether the global transcriptional changes in SMC1A-R586W mESCs resulted in functional changes to those cells. We assessed the potential of cells to differentiate into embryoid bodies (EBs), an *in vitro* process which mimics the *in vivo* process of gastrulation and formation of germ layers [42]. We triggered EB formation by culturing a fixed number of wildtype or SMC1A-R586W mESCs in hanging droplets of media lacking leukemia inhibitory factor (LIF). SMC1A-R586W mESCs form smaller EBs than wildtype cells (**S1H Fig**). This may be explained in part by proliferation differences, as SMC1A-R586W mESCs grew more slowly than wildtype cells (**S1I Fig**). Notably, the proliferation differences are not explained by delays in a specific phase of the cell cycle, as asynchronously growing wildtype and SMC1A-R586W mESCs show similar percentages of cells in G1, S, and G2/M phases of the cell cycle (**S1J Fig**). We further assessed differentiation by transferring EBs from hanging-droplet cultures to tissue-cultured-treated dishes and allowing them to continue differentiating in media lacking LIF. Under these conditions, 5–20% of cells in the population develop into beating cardiomyocytes, which can be observed with light microscopy [43]. We monitored wildtype and SMC1A-R586W cultures for 10 days and noted that mutant EB cultures lagged in both first appearance of beating cells and in total number of wells that ultimately exhibit beating (**S1K Fig**). Together, these data indicate that the SMC1A-R586W mutation substantially alters gene expression in mESCs and disrupts their maintenance of pluripotency and differentiation into other cell types.

### Minimal disruption of active or repressive histone modifications in SMC1A-R586W mESCs

**Fig 2:**
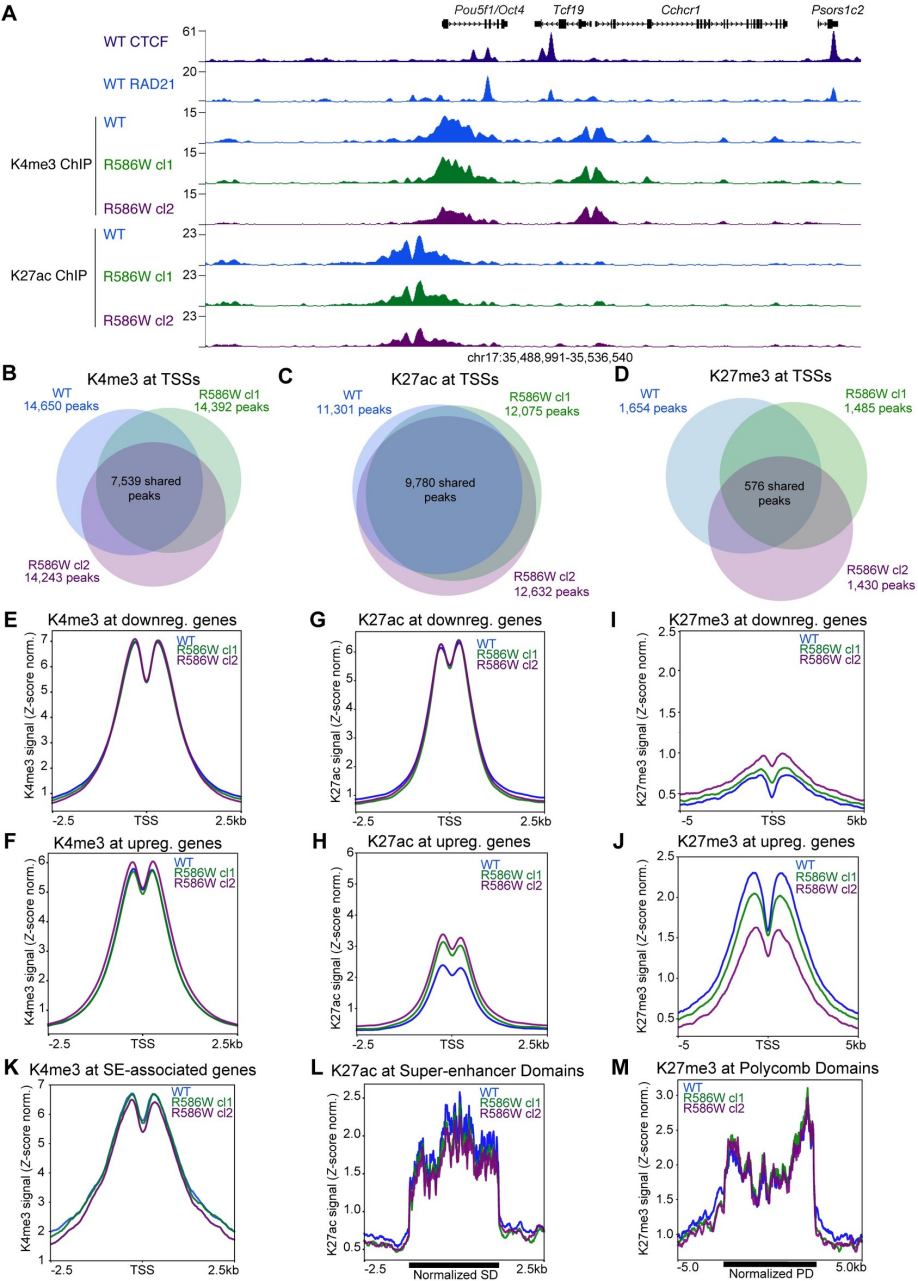
Active histone markings at promoters and enhancers are similar between wildtype and SMC1A-R586W mESCs. **A** UCSC Genome Browser coverage tracks showing K4me3 and K27ac distribution at the *Pou5f1 (Oct4)* locus in mESCs. CTCF data were previously published [68]. **B** Overlap of K4me3-marked TSSs, **C** K27ac-marked TSSs, and **D** K27me3-marked TSSs in wildtype and SMC1A-R586W mESCs. **E** K4me3 signal at the TSSs of genes downregulated and **F** upregulated in SMC1A-R586W mESCs. **G** K27ac signal at the TSSs of genes downregulated and **H** upregulated in SMC1A-R586W mESCs. **I** K27me3 signal at the TSSs of genes downregulated and **J** upregulated in SMC1A-R586W mESCs. **K** K4me3 signal at TSSs of Super-enhancer associated genes. **L** K27ac signal at Super-enhancer Domains. **M** K27me3 signal at Polycomb Domains identified by Dowen et al. (2014). K4me3 and K27me3 data represents merge of two biological replicates for each group. K27ac data shown represents merge of three biological replicates of wildtype and two each of SMC1A-R586W clone 1 and SMC1A-R586W clone 2.

Gene activity is linked to the histone code, such that actively transcribed regions and sites of transcriptional repression are marked by distinct histone tail modifications. Aberrant changes in histone modifications and chromatin state are associated with cancer [44]. Therefore, we sought to investigate whether the SMC1A-R586W mutation may alter the pattern of histone modifications, and thereby, contribute to differential expression of genes. We assessed whether the chromatin landscape was altered by performing chromatin immunoprecipitation followed by high-throughput sequencing (ChIP-seq) for histone 3 lysine 4 trimethylation (K4me3), lysine 27 acetylation (K27ac), and lysine 27 trimethylation (K27me3) in wildtype and mutant mESCs. Surprisingly, we found that global K4me3, K27ac, and K27me3 levels were largely unchanged between wildtype and SMC1A-R586W mESCs (**Figs 2A and S2A**). Peak calling identified thousands of K4me3, K27ac, and K27me3 sites in wildtype and mutant mESCs, with many peaks of each modification overlapping with TSSs (**Figs 2B–2D and S2B–S2D**). Peaks in SMC1A-R586W mESCs exhibited partial overlap with wildtype peaks for all three histone modifications. We also examined whether these histone modifications were altered at differentially expressed genes, and found no changes in K4me3 at DEGs that were up- or downregulated in SMC1A-R586W (**Fig 2E and 2F**). Modest increases in K27ac and decreases in K27me3 were observed at TSSs of upregulated genes, consistent with the increased transcriptional activity at these genes (**Fig 2G and 2J**). No substantial changes in histone modifications were observed at SE-associated or Polycomb-repressed genes (**Fig 2K and 2M**). These results suggest that the the differentially-expressed genes in SMC1A-R586W mESCs are not well-correlated with changes in histone modifications, and instead the mutant cohesin hinge domain may impact other mechanisms of gene regulation.

We used the Diffbind software package to assess quantitative differences in histone modifications at a set of consensus sites across the genome [45]. While we found few quantitative changes in K4me3 or K27me3 levels, we did observe over 3,000 K27ac peaks that significantly gained signal in SMC1A-R586W mESCs (**S2E and S2G Fig**). Of these, over 800 were found at a TSSs associated with an upregulated DEG, identifying a class of sites where gene expression changes were correlated with histone modification changes in SMC1A-R586W mESCs. We identified classes of K4me3, K27ac, and K27me3 peaks that were unique to either WT or SMC1A-R586W mESCs and overlapped TSSs of differentially expressed genes. We saw that genes whose TSSs gained K4me3 or K27ac peaks in the SMC1A-R586W mESCs tend to increase in expression (**S2H and S2I Fig**). However, loss of a K27me3 peak from a TSS in the SMC1A-R586W mutant was not associated with increased gene expression (**S2J Fig**). Together, these data demonstrate that levels of histone modifications associated with active and repressive chromatin states are largely unchanged in SMC1A-R586W mESCs, and those changes that do occur are well-correlated with changes in gene expression. Therefore, we conclude that misregulation of chromatin state is not a major driving force of the gene expression changes observed in SMC1A-R586W mutant cells.

### Reduced cohesin at promoters and enhancers in SMC1A-R586W mESCs

**Fig 3:**
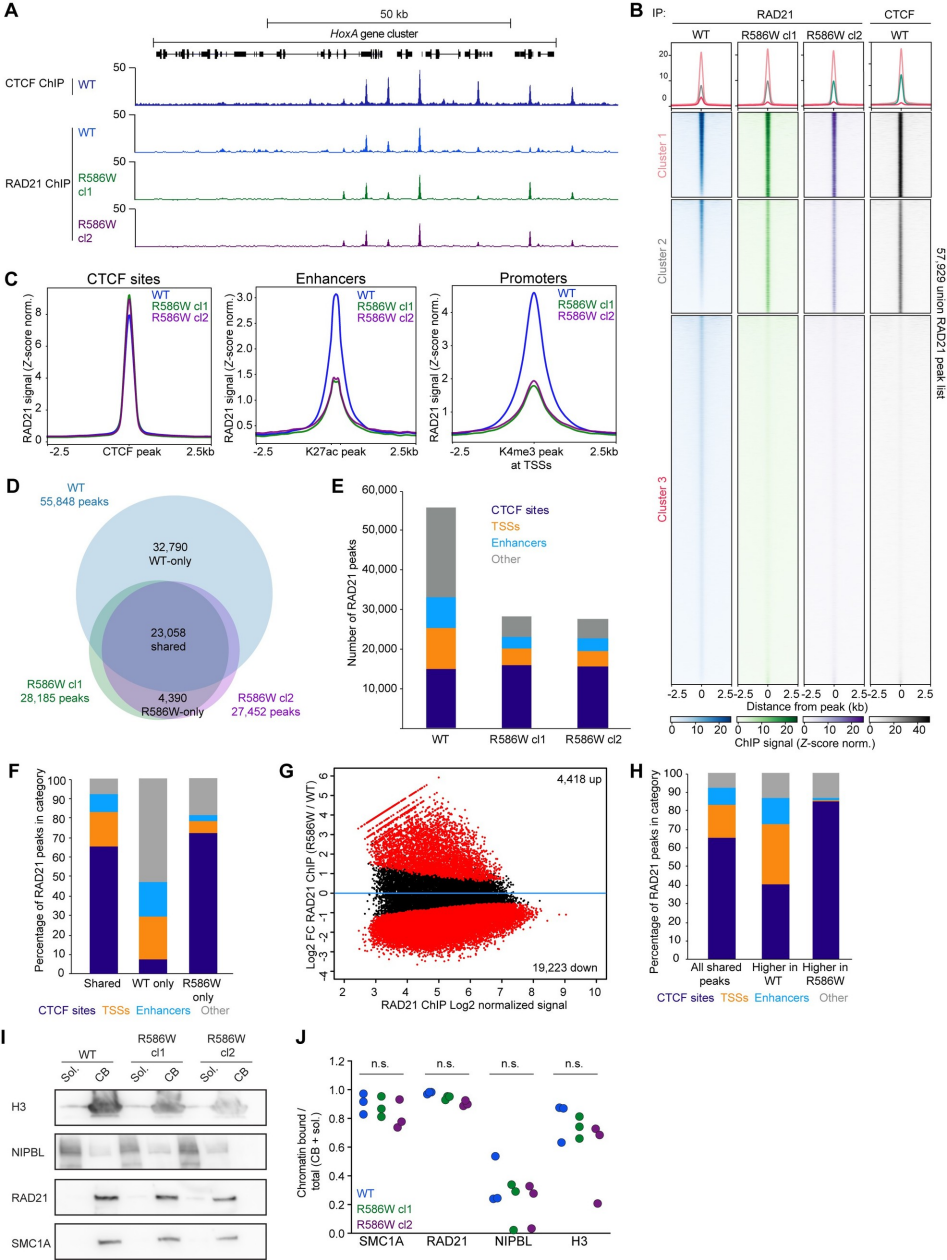
Cohesin is selectively depleted at promoters and enhancers in SMC1A-R586W mESCs. **A** RAD21 enrichment measured by ChIP-seq at the *HoxA* locus in wildtype and R586W cl1 and cl2. **B** Heatmap representation of RAD21 and CTCF enrichment, with k-means clustering. Rows are ranked in descending order based on WT RAD21 signal (column 1). **C** Average signal plots depicting RAD21 enrichment at CTCF sites, a merged list of K27ac peaks (enhancers), and a merged list of K4me3-marked TSSs (promoters) in wildtype and R586W cl1 and cl2. **D** Overlap of called peaks in wildtype and R586W cl1 and cl2. **E** Distribution of RAD21 peaks across the given genomic sites in wildtype and R586W mESCs. **F** Distribution of RAD21 peaks common to both R586W clones but not wildtype mESCs. **G** Differential analysis using Diffbind of RAD21 signal at peaks common to wildtype and both R586W clones. Red, p-adj<0.1. **H** Distribution across given genomic sites of peaks with increased or decreased signal in R586W mESCs as measured by Diffbind. For analyses shown, data from 2 biological replicates for each of wildtype, R586W cl1, and R586W cl2 were merged. **I** Representative western blots showing the levels of indicated proteins in soluble (Sol.) and chromatin-bound (CB) fractions. **J** Amounts of chromatin-bound protein expressed as a fraction of total (chromatin-bound + soluble) signal for WT, R586W cl1, and R586W cl2. n = 3 biological replicates. All differences not significant as measured by two-way ANOVA. Numerical data are presented in **S6 Table**.

To investigate possible mechanisms by which the SMC1A-R586W mutation might alter cohesin function in order to cause global transcriptional changes, we evaluated cohesin distribution on chromatin by performing ChIP-seq for the core cohesin subunit RAD21. Given that other hinge mutations characterized in yeast impair cohesin loading, we hypothesized that the SMC1A-R586W mutation might act similarly and cause a global reduction in cohesin signal. ChIP-seq data were of good quality, and showed strong correlation between replicates and between the two SMC1A-R586W clones (**S3A-S3E Fig and S2 Table**). Whereas global RAD21 levels were generally maintained in mutant cells compared to wildtype (**Fig 3A and 3B**), at promoters and enhancers there was a strong reduction in RAD21 signal (**Fig 3C**). Additionally, both mutant clones showed reduced signal-to-noise compared to wildtype (**S3C and S3D Fig**). K-means clustering of RAD21 signal into three distinct categories was performed: a group of high-amplitude RAD21 signal in all groups marked also by high CTCF signal in wild type cells; a group with relatively higher RAD21 signal in SMC1A-R586W mESCs marked by intermediate wildtype CTCF signal, and a large group with low-amplitude RAD21 signal in wild type, minimal RAD21 signal in the two mutant lines, and minimal CTCF signal in wild type cells (**Fig 3B**). Peak calling using merged replicates revealed fewer peaks in SMC1A-R586W compared to wildtype mESCs (~28,000 versus 52,000, respectively) (**Fig 3D**). The number of RAD21 peaks overlapping CTCF sites was largely unchanged in SMC1A-R586W mESCs, while overlap of RAD21 peaks with K4me3-marked promoters and K27ac-marked enhancers was substantially decreased (**Fig 3E**). The 4,390 ectopic RAD21 peaks gained in both SMC1A-R586W clones (but not present in wildtype mESCs) mostly overlapped CTCF sites, while RAD21 peaks specific to wild type mESCs more frequently overlapped promoters and enhancers and rarely overlapped CTCF sites (**Fig 3F**). Similarly, differential binding analysis of a consensus peak list revealed a relatively small number of sites with increased RAD21 signal in SMC1A-R586W mESCs compared to wildtype, which tended to overlap CTCF sites, and a larger set of sites with decreased RAD21 signal that tended to overlap promoters and enhancers (**Fig 3G and 3H**). Furthermore, RAD21 loss was broadly observed across sites with active histone modifications, including K4me3-marked TSSs, and both TSS-proximal and -distal K27ac peaks (**S3F and S3H Fig**). Given the large set of genes whose expression either increases or decreases in R586W mESCs, we investigated cohesin levels at their promoters. Strikingly, RAD21 was similarly depleted from the promoters of both upregulated and downregulated genes in SMC1A-R586W mESCs, indicating that cohesin may be required for both activation and repression of gene expression (**S3I and S3J Fig)**. Finally, given that the SMC1A-R586W mutation causes loss of cohesin from a subset of genomic sites, we performed subcellular protein fractionation experiments to assess bulk cohesin levels on the genome (**Fig 3I and 3J**). These results revealed that neither the core cohesin complex or loading subunit NIPBL are depleted from chromatin in bulk as a consequence of the SMC1A-R586W mutation. We conclude that the SMC1A-R586W mutation interferes with cohesin localization to promoters and enhancers, and that this interference results in the dysregulated transcription observed in mutant cells.

### Reduction of small DNA loops in SMC1A-R586W mESCs

**Fig 4:**
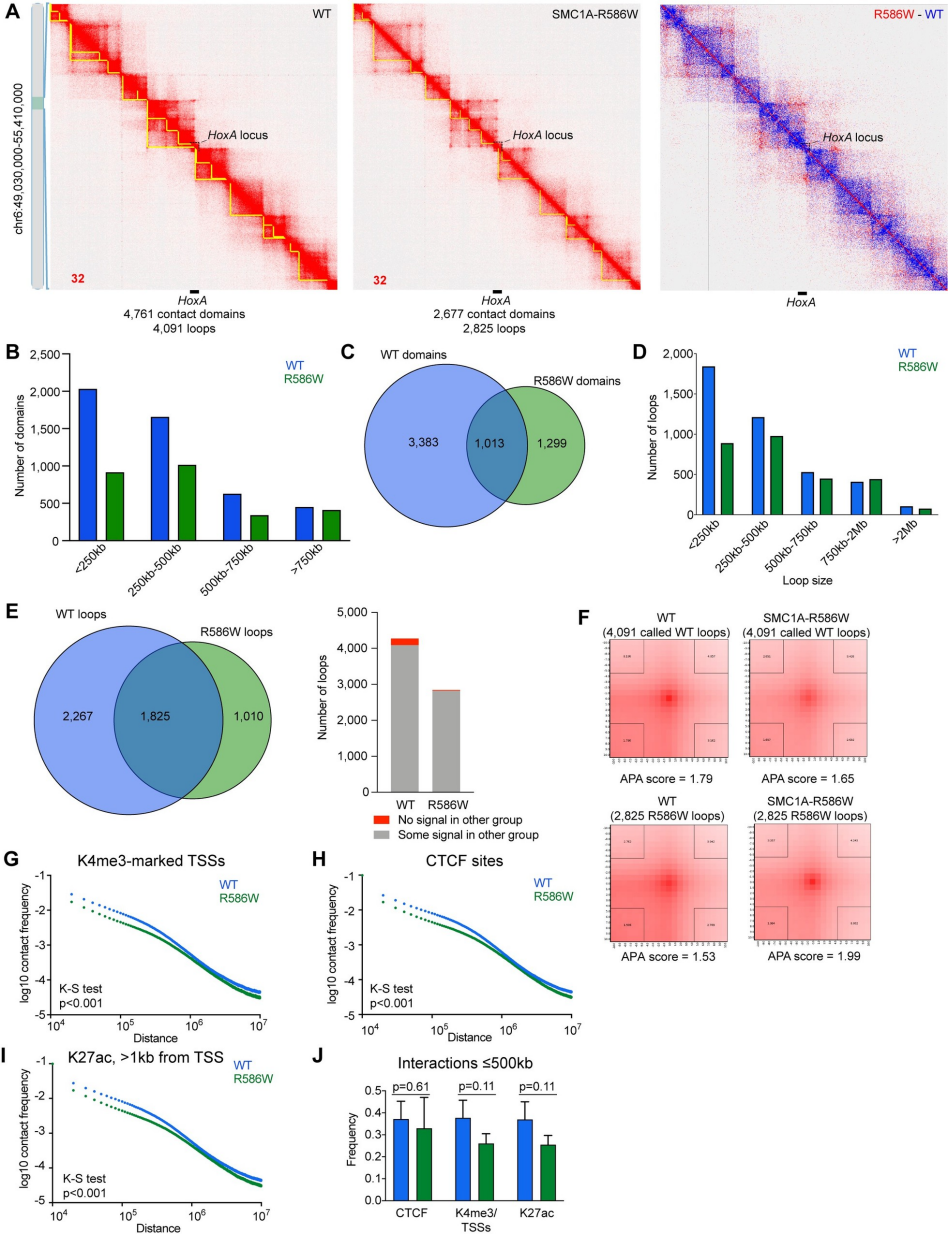
Altered chromatin looping in SMC1A-R586W mESCs. **A** Hi-C heatmaps at 10kb resolution centered around the *HoxA* locus in wildtype and R586W mESCs. Yellow lines indicate positions of contact domains called by Arrowhead in each. **B** Distribution of contact domains by size in WT and SMC1A-R586W mESCs. **C** Overlap of domain positions in WT and SMC1A-R586W mESCs. **D** Distribution of loops by size in WT and SMC1A-R586W mESCs. **E** Left, overlap of loop positions between wildtype and R586W mESCs as measured by Juicer Tools CompareLists routine. Right, overlap of loop positions as measured by Juicer Tools HICCUPsDiff routine **F** Aggregate peak analysis (APA) in wildtype and R586W mESCs, at wildtype loops (top row) or R586W loops (bottom row). p<0.05 as measured by Kruskal-Wallis test with Dunn’s multiple comparisons correction for wildtype vs R586W when APA is determined at wildtype loop calls (top row), and wildtype vs R586W when APA is determined at R586W loop calls (bottom row). **G** Distribution of contact distances with K4me3-marked TSSs, **H** CTCF sites, or **I** TSS-distal K27ac sites in WT and SMC1A-R586W mESCs. Line is mean of two replicates in each group. Statistical significance tested using Kolmogorov-Smirnoff test. **J** Binned interactions under 500kb with given genomic feature. Bars represent mean±s.d. for each group. Differences n.s. as measured by two-way ANOVA.

Since the SMC1A-R586W mutation reduces cohesin levels at promoters and enhancers, we investigated whether chromatin looping was also altered. We performed in situ Hi-C on wildtype and SMC1A-R586W mESCs, and analyzed the data using the Juicer software package [46]. We generated two Hi-C replicates from wildtype and SMC1A-R586W mESCs, totaling approximately 1.1x10^9^ reads for each genotype, and determined that the data were of good quality and replicates were well-correlated using several metrics (**S4A and S4B Fig and S4 Table**). Reads from replicates were merged together for subsequent analyses. The Arrowhead algorithm was used to identify contact domains, which represent regions that contain many Hi-C contacts. We found a striking depletion of short-range, intra-domain contacts in SMC1-R586W mESCs at the *HoxA* locus (**Fig 4A**). We further compared genome organization, histone modifications, and cohesin occupancy at two AML tumor suppressor genes identified as downregulated in SMC1A-R586W mESCs. *Dnmt3a* expression was reduced by >70% in SMC1A-R586W mESCs, and Hi-C revealed that local, short-range contacts around the *Dnmt3a* locus are substantially decreased, with several small contact domains observed in wildtype and two larger contact domains present in SMC1A-R586W mESCs (**S5A Fig**). Notably, RAD21 ChIP-seq signal is substantially reduced in both SMC1A-586W mESC clones compared to wildtype, while K27ac signal is not altered (**S5B Fig**). A similar pattern is observed at the tumor suppressor and Super-enhancer Domain gene *Tet2*, whose expression is decreased by 65% in SMC1A-R586W mESCs. Depletion of local contacts are observed at the *Tet2* locus, yet no change in K27ac signal is observed (**S5C and S5D Fig**). Additionally, RAD21 signal is largely maintained at CTCF sites flanking the *Tet2* gene, but is lost from the *Tet2* TSS in SMC1A-R586W mESCs. These results suggest that loss of cohesin from critical disease-associated loci may cause loss of regulatory DNA contacts and alter gene expression.

Fewer contact domains were called in SMC1A-R586W mESCs relative to wildtype, with smaller contact domains (<500kb in size) exhibiting the greatest loss (**Figs 4B and 4C and S4C**). Insulation scores and directionality indices were measured to assess the strength of contact domains and their ability to prevent contacts from crossing their boundaries [47,48]. These analyses revealed that SMC1A-R586W contact domains were less defined and exhibited reduced insulation capacity compared to wildtype (**S4D and S4E Fig**).

Next, we used the HiCCUPs algorithm to identify DNA loops, which are pairwise contacts between distal regions of the genome. Similar to our analysis of contact domains, we detected fewer DNA loops in SMC1A-R586W mESCs than wildtype and found that the distribution was shifted toward longer loops in the mutant cells (**Figs 4D and 4E and S4**). This loss of shorter DNA loops in SMC1A-R586W mESCs is consistent with a loss of enhancer-promoter contacts (**Fig 4D**). A simple intersection of loop anchor positions identified sets of loops lost and gained in SMC1A-R586W mESCs, however, HiCCUPsDiff analysis demonstrated that virtually all gains and losses represent quantitative changes in loop strength, not qualitative changes to loop structures (**Fig 4E**). Because most wildtype loops are retained in SMC1A-R586W cells, just at sub-threshold levels, we performed Aggregate Peak Analysis (APA) [49] using both the wildtype loop list and SMC1A-R586W loop list to quantify the changes in loop strength. Excluded from this analysis were relatively rare, but extremely large loops (> 2Mb) as they may represent technical artifacts of the loop-calling algorithm and not reflect biologically meaningful structures. We found that DNA loops identified in wildtype mESCs displayed a significantly stronger APA score in wildtype than SMC1A-R586W cells (**Fig 4F**). Interestingly, the reciprocal analysis was also true, DNA loops identified in SMC1A-R586W cells displayed a significantly stronger APA score in SMC1A-R586W cells than in wildtype. Since APA score is determined by measuring the enrichment of signal in the point of a called loop relative to signal in the interior of the loop [49], this analysis may reflect the diminished intra-loop signal in SMC1A-R586W cells compared to wildtype rather than enhanced strength of a DNA loop boundary. Finally, we investigated whether the strength of DNA loops related to loop size by performing APA on the wildtype DNA loop list subsetted into the size categories presented in **Fig 4D**. This analysis revealed that SMC1A-R586W cells have weaker DNA loops across all size categories compared to wildtype (**S4G Fig**).

To further explore whether enhancer-promoter loops were specifically lost in SMC1A-R586W mESCs, we overlapped the positions of loop anchors in each group with promoter and enhancer elements (**Fig 2**) and saw that loops anchored by K4me3-marked TSSs or TSS-distal K27ac sites were more frequently lost than gained in SMC1A-R586W mESCs relative to wild type (**S4H Fig**). Conversely, SMC1A-R586W mESCs were better able to retain loops anchored by CTCF and RAD21 (**S4I Fig**). We evaluated more transient interactions formed by promoters and enhancers by plotting the frequency distributions of contact lengths with promoters, distal K27ac sites, and CTCF-bound elements and noted a striking depletion of short-range contacts at all three classes in SMC1A-R586W mESCs (**Fig 4G and 4J**). Finally, we assessed whether the organization of the genome into active and repressed compartments was altered by the SMC1A-R586W mutation, as loss of Cohesin activity has been associated with altered compartment structure [50]. Minimal changes in compartmentalization of the genome were observed in SMC1A-R586W mESCs compared to wildtype (**S4J and S4K Fig**). We conclude that the SMC1A-R586W mutation impairs the ability of mESCs to organize chromatin into loops and contact domains, which correspond to short-range contacts between regulatory elements such as enhancers and promoters.

### Relationship between SMC1A-R586W and cohesin accessory proteins

**Fig 5:**
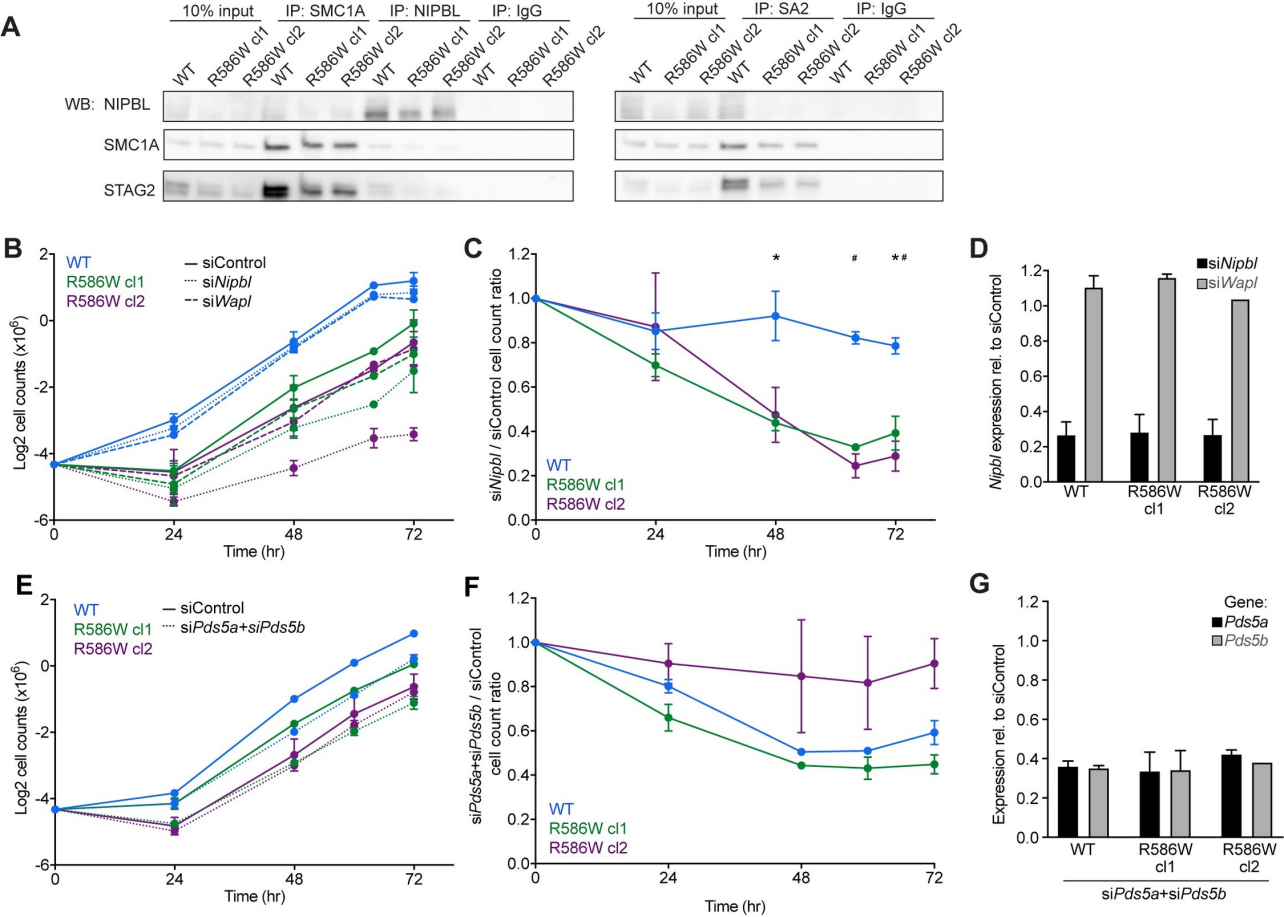
Physical and functional interactions between NIPBL and Cohesin in SMC1A-R586W mESCs. **A** Co-immunoprecipitation experiments were performed in wildtype and SMC1A-R586W mESCs with the indicated antibodies. Western blots shown are representative of 3 independent biological replicates. **B** Log2-transformed cell counts at the given time points in wildtype and SMC1A-R586W mESCs transfected with the indicated siRNA. n = 4 biological replicates for siControl and si*Nipbl*, and N = 2 biological replicates for si*Wapl*. **C** Ratio of si*Nipbl* to siControl proliferation in wildtype and SMC1A-R586W mESCs. *, p<0.05 as measured by two-way ANOVA between WT and R586W cl1. #, p<0.05 as measured by two-way ANOVA between WT and R586W cl2. **D** Transcript levels in si*Nipbl* and si*Wapl* transfected mESCs. n = 4 biological replicates for si*Nipbl*, n = 2 for WT and R586W clone 1 si*Wapl*, and n = 1 for R586W clone 2 si*Wapl*. p<0.05 as measured by two-way ANOVA. **E** Log2-transformed cell counts at the given time points in wildtype and SMC1A-R586W mESCs transfected with the indicated siRNA. n = 2 biological replicate for siControl and si*Pds5a+Pds5b*. **F** Ratio of si*Pds5a+Pds5b* to siControl proliferation in wildtype and SMC1A-R586W mESCs. n = 2 biological replicates. All differences not significant as measured by two-way ANOVA. **G** Transcript levels in si*Pds5a+Pds5b* knockdown mESCs expressed relative to siControl as measured by RT-qPCR. n = 2 biological replicates for all groups. Numerical data are presented in **S6 Table**.

Given recent findings that the R586 region of SMC1A may interact with STAG proteins and NIPBL [13], we sought to investigate whether the SMC1A-R586W mutation disrupts the physical or functional interactions between the core cohesin complex and NIPBL or STAG proteins. To assess this, we performed coIPs with antibodies targeting SMC1A, NIPBL, and STAG2 in wildtype and SMC1A-R586W mESCs (**Fig 5A**). Western blotting on precipitated proteins revealed that incorporation of STAG2 and NIPBL into the cohesin complex was largely unchanged between wildtype and SMC1A-R586W mESCs (**Figs 5A and S6A–S6E**). Next, we assessed whether functional interactions between the cohesin complex and its accessory proteins were impacted by the SMC1A-R586W mutation. Since NIPBL is required for cohesin loading at promoter and enhancer elements [51–53], we investigated whether wildtype and SMC1A-R586W cells are similarly impacted by *Nipbl* knockdown. *Nipbl* knockdown using siRNA dramatically reduced cellular proliferation of SMC1A-R586W, but not wildtype mESCs (**Figs 5B–5D and S6F**), indicating greater sensitivity to NIPBL loss in the mutant cells. In other cell types, depletion of the cohesin unloading factor WAPL increases the amount of cohesin on chromatin and rescues cell growth phenotypes caused by reduced NIPBL function [54]. Therefore, we investigated whether knockdown of WAPL rescues the reduced proliferation of SMC1A-R586W mESCs. Instead, *Wapl* knockdown failed to increase proliferation of SMC1A-R586W mESCs (**Figs 5B, and S6E–S6H**), suggesting that the defect in SMC1A-R586W mESCs is not fully explained by disruption of NIPBL-mediated cohesin loading. In yeast, Pds5 potently inhibits Scc2/NIPBL activity, arresting cohesin translocation and ATP hydrolysis [32]. Therefore, we investigated whether co-depletion of PDS5A and PDS5B might rescue proliferation defects in SMC1A-R586W mESCs. We performed SMC1A coIP and confirmed that PDS5A and PDS5B are capable of incorporation into the cohesin complex in SMC1A-R586W mESCs (**S6I Fig**). However, simultaneous treatment of mESCs with *Pds5a* and *Pds5b* siRNAs caused similar proliferation defects in wildtype and SMC1A-R586W cells (**Figs 5E–5G and S6J**). Together, these data indicate that the proliferation defect of SMC1A-R586W mESCs is not simply due to altered cohesin levels on the genome, since it is largely independent of known cohesin loading and unloading processes.

## Discussion

Here, we demonstrate that a cancer-associated mutation to the hinge domain of SMC1A results in striking changes to gene expression and genome organization. Cohesin complexes containing SMC1A-R586W show reduced occupancy at enhancers and promoters, and decreased short-range DNA loops. The DNA loops and contact domains formed in SMC1A-R586W mESCs are compromised, as measured by several metrics. These results suggest that the SMC1A-R586W mutation interferes with both cohesin localization and the ability of cohesin complexes to mediate DNA loop interactions. These changes in DNA loop interactions are sufficient to alter gene expression even in the absence of large-scale changes to active or repressive chromatin modifications. Notably, the broad transcriptional and architectural changes observed in SMC1A-R586W mESCs occur in the absence of additional cancer mutations, single nucleotide polymorphisms, and variants seen in disease settings, demonstrating that altered cohesin function is sufficient to bring about changes that may contribute to the disease state.

Despite the changes in gene expression observed in SMC1A-R586W mESCs, there were not major changes in H3K27ac, H3K4me3 and H3K27me3 patterns, suggesting a partial uncoupling of the histone code and gene expression. Rather than causing changes to the histone code, the SMC1A-R586W mutation may impact RNA polymerase II (RNAPII) elongation. Enhancers are thought to stimulate the transition of the paused form of RNAPII to the elongating form that productively synthesizes RNA [55,56]. Indeed, cohesin depletion or cohesin mutations have previously been shown to increase RNAPII pausing at cohesin-bound genes and decrease transcription in gene bodies [57]. Therefore, cohesin can act like other enhancer-bound proteins that promote RNAPII elongation through gene bodies leading to productive transcription. Our results are consistent with studies demonstrating that altered levels of cohesin subunits lead to significant changes in expression of hundreds to thousands of genes [38,58,59]. This is somewhat different from reports where acute or transient depletion of cohesin subunits causes relatively fewer genes to change in expression [50,60]. Interestingly, while acute depletion of RAD21 levels by approximately 80 percent only changes expression of dozens of genes, it is sufficient to reduce local, intra-domain contacts, similar to the pattern observed in SMC1A-R586W mESCs [60]. We also note that, despite the broad transcriptional changes observed in SMC1A-R586W mESCs, they exhibit minimal changes in compartmentalization. This is consistent with the relatively unchanged histone modification landscape in SMC1A-R586W mESCs, as compartmentalization is driven in part by chromatin state [61]. Instead, transcriptional changes in SMC1A-R586W appear to be driven by a decrease in DNA loops and contact domains, without major changes in chromatin state.

These findings represent the first functional characterization of the cohesin hinge in mammalian cells and reveal a role for the hinge domain in regulating cohesin localization and chromatin looping. In yeast, suppressor screens have implicated the hinge in both establishing the topological embrace of DNA required for sister chromatid cohesion and in permitting non-topological interactions with DNA [31,33,34]. Neutralization of positively charged Smc1 and Smc3 residues near the central pore of the hinge prevents topological association with chromatin, while mutation of residues that destabilize the Smc1-Smc3 hinge interface abrogates both topological and non-topological interactions. While the R586W mutation does disrupt a positively charged residue similar to the yeast mutations in the pore of the hinge domain, we note several distinctions between the R586W and yeast mutant phenotypes. The R586 residue is located far from the central pore structure and the SMC1A-SMC3 hinge interface, and the absence of cell cycle defects in SMC1A-R586W mESCs suggests that the mutation does not impair topological engagement of cohesin with chromatin, as was shown with several yeast hinge mutations [31]. Our ChIP-seq data further indicate that cohesin loading is not affected to the extent required to disrupt sister chromatid cohesion and the cell cycle [62]. Therefore, it is unlikely that SMC1A-R586W disrupts the central pore or stability of the hinge domain.

Our evidence indicates that the R586W mutation alters cohesin localization and DNA looping, potentially by altering interaction between the hinge and cohesin accessory proteins or DNA. Several studies of yeast cohesin suggest the hinge interacts with both the kleisin-bound accessory proteins STAG and PDS5, and the ATPase heads of SMC1 and SMC3 [6,63,64]. Xu et al. (2018), employed suppressor screens in yeast that indicated the cohesin hinge may cooperate with the ATPase/kleisin/HEAT interface to dynamically bind and release DNA as part of DNA loop extrusion by the complex [29,30,36]. This dynamic binding and release of chromatin is thought to involve a conformational change of cohesin between extended and folded states, resulting in an inchworm-like motion [36]. Cryo-EM structures suggest that the cohesin hinge may make dynamic contacts with STAG1, single stranded DNA, or NIPBL through residues including R586 [13]. However, depletion of PDS5A and PDS5B or WAPL failed to rescue the SMC1A-R586W-induced proliferation defect, indicating that decreased cohesin unloading is not sufficient to rescue the mutant phenotype. This suggests that the SMC1A-R586W defects are independent of NIPBL and its function as a cohesin loader. Thus, the R586W mutation may instead disrupt the folded conformation of cohesin and thereby reduce the efficiency of cohesin motion or loop extrusion. While our work does not directly address involvement of the cohesin hinge in translocation or DNA loop extrusion, our Hi-C results reveal a loss of intra-domain contacts similar to that seen upon inhibition of cohesin ATPase activity in lymphoblastic cell lines [28]. However, whereas inhibition of cohesin ATPase activity caused a selective decrease in cohesin signal at CTCF sites, the SMC1A-R586W mutation caused a selective decrease in cohesin signal at enhancers and promoters. This suggests that both the hinge and the ATPase domains of cohesin play important, yet distinct roles in cohesin mobility on chromatin.

Notably, our results are the first to demonstrate that a cancer mutation in the cohesin complex causes changes in genome organization. In murine AML models, cohesin point mutations and loss-of-expression mutations have been shown to participate in leukemogenesis by permitting hematopoietic stem cells (HSCs) to explore aberrant, pre-leukemic transcriptional programs and open novel enhancer-like elements in chromatin [21–24]. However, these studies do not establish a direct mechanism through which mutant cohesin molecules drive disease pathology. While we observe similar transcriptional dysregulation in our SMC1A-R586W mESCs, Hi-C data indicate that SMC1A-R586W may act primarily through weakening of intra-domain regulatory contacts. Similar loss of local chromatin interactions was observed in *Stag2*-null murine HSCs, suggesting that this finding may be generalizable to a number of pathologies linked to cohesin mutations [65].

While cancer cells are generally thought to grow and divide rapidly, the SMC1A-R586W mutation causes decreased proliferation of mESCs. A possible explanation for how this genetic mutation may contribute to oncogenesis, independent of control of cell growth rate, is through an epigenetic mechanism. Cohesin is an epigenetic regulator of gene expression and cellular identity. The aberrant cell identity in SMC1A-R586W mESCs may reflect the ‘epigenetic priming’ effect described for several first-hit mutations in myeloid neoplasia [66]. Reduced or ablated activity of epigenetic regulators such as DNMT3A and TET2 causes precocious expression of differentiation-associated gene expression programs in HSCs; additional mutations cause unchecked proliferation resulting in overt oncogenesis. Similar phenotypes emerge in models of cohesin cancer mutations during murine hematopoiesis [21,22,65]. Cohesin mutations in cancer may permit cancer-initiating cells to explore aberrant developmental fates before acquiring other mutations that cause hyper-proliferative phenotypes. The hematopoietic system may be especially sensitive to such mutations in epigenetic regulators, as HSCs turn over slowly and a clonal subpopulation with increased self-renewal capacity could potentially survive for decades before acquiring the additional mutations needed for myeloid neoplastic syndromes to emerge [67]. Consistent with this, in a murine model of AML driven by monoallelic inactivation of *Smc3*, disease only developed once *Smc3*^+/-^ HSCs were also given the proliferation-driving FLT3-internal tandem duplication (ITD) cancer mutation, although SMC3 loss alone was sufficient to cause aberrant activation of non-HSC transcriptional programs [23]. Conversely, SMC1A-R586W may represent a second hit that creates conditions permissive to oncogenesis, where benign neoplasias can adopt more aggressive characteristics. We propose that a similar mechanism is at work in SMC1A-R586W cancers, and that this multi-hit model may be generalizable to other cohesin loss-of-function mutations in a variety of cancers.

## Methods

### Antibodies

For western blotting, polyclonal primary antibodies against SMC1A (Bethyl Laboratories, Montgomery, TX; A300-055A), SMC3 (Abcam, Cambridge, MA; ab9263), RAD21 (Bethyl Laboratories, A300-080A), STAG1 (Bethyl Laboratories, A300-157A), STAG2 (Bethyl Laboratories, A300-158A), PDS5A (Bethyl Laboratories, A300-089A), PDS5B (Bethyl Laboratories, A300-538A), and NIPBL (Bethyl Laboratories, A301-079A) were used. Secondary horseradish peroxidase-conjugated antibodies raised against rabbit immunoglobulin (IgG) in donkey (GE Healthcare, Chicago, IL; NA934) or against goat IgG in rabbits (Abcam, ab97100) were used. For ChIP, polyclonal antibodies against RAD21 (Bethyl Laboratories, A300-080A), H3K4me3 (Abcam, ab8580), H3K27ac (Active Motif, Carlsbad, CA; 39135), H3K27me3 (Abcam, ab6002) were used. For CoIP, polyclonal antibodies against SMC1A (Bethyl Laboratories, A300-055A), NIPBL (Bethyl Laboratories, A301-079A), STAG2 (Bethyl, Laboratories A300-159A) and non-specific polyclonal rabbit IgG (Bethyl Laboratories, P120-101) were used.

### Cell lines

V6.5 murine embryonic stem cells were a gift from R. Young of the Whitehead Institute for Biomedical Research. V6.5 are male cells derived from a C57BL/6(F) x 129/sv(M) cross.

### Cell culture

Naïve V6.5 mESCs were grown as previously described [68]. To assess proliferation, cells were seeded at an initial density of 5 x 10^4^ cells in wells of a six-well dish, and counts were collected at 24, 48, either 60 or 64, and 72 hours after seeding. To count, cells were trypsinized and resuspended in PBS, then mixed with trypan blue and counted as above. Wells were set up in triplicate at each time point.

mESCs were differentiated into EBs using hanging droplet cultures as described [69], with modifications. Briefly, mESCs were dissociated into single-cell suspension and placed into hanging droplets in the same media as before, lacking LIF supplementation. After three days, images were acquired using an EVOS FL light microscope (Thermo Fisher). For further differentiation, individual droplets were transferred to gelatinized wells of 24-well plates and maintained in the same media as before. Development of beating cardiomyocytes was monitored by daily examination under a microscope.

For cell cycle analysis, cells growing in asynchronous cultures were placed in single-cell suspension, then fixed and stored in 70% ethanol at -20°C. To assess DNA content, cells were incubated with 100μg of RNAse A for 2 hours, then stained with 50μg/ml propidium iodide in PBS. Data were acquired on a BD FACScan instrument. For analysis, FlowJo software (TreeStar) was used to gate live, single cells using forward and side scatter, and cell cycle status was determined by using propidium iodide signal to establish the fraction of cells with 2N (G0/G1), intermediate (S), or 4N (G2/M) chromsomes.

### Genome editing

Genome-edited mESC lines weres generated as previously described [68], with modifications. mESCs were transfected with a single-stranded donor oligonucleotide (ssODN) repair template, plasmids encoding a synthetic guide RNA (sgRNA), Cas9 and a fluorescent gene (eGFP or mCherry) using Lipofectamine 2000 (Thermo Fisher, 11-668-027). Single GFP^+^RFP^+^ cells were sorted by UNC Flow Cytometry Core Facility staff using a FACSAria II (BD Biosciences, San Jose, CA). Fluorescent cells were grown in dilute cultures on irradiated murine embryonic fibroblast monolayers to form colonies. Individual colonies were expanded into clonal cell lines, screened for genome edits by PCR and Sanger sequencing, and cryogenically stored. Individual allele sequences were determined by PCR of the region surrounding the mutated site, followed by TOPO-TA cloning (Thermo Fisher, K4575J10) and Sanger sequencing. sgRNA and ssODN sequences are provided below and were designed using the CRISPR tool (crispr.mit.edu). Both SMC1A-R586W clones bear a c.1756C>T mutation in the *Smc1a* gene, resulting in a codon change from CGG to TGG. Clone 1 is known as *Smc1a*^*tm1(c*.*1756C>T)Jdow*^, according to Jackson Laboratories Mouse Genome Informatics nomenclature conventions. Clone 2 contains an additional mutation, c.1770G>C, that inactivates the protospacer adjacent motif (PAM) and causes a silent codon change, and is known as *Smc1a*^*tm2(c*.*1756C>T*,*c*.*1770G>C)Jdow*^.

sgRNA 1: 5’- CACCGAGAAACTCCGGGAGCTGAAG -3’

sgRNA 2: 5’- AAACCTTCAGCTCCCGGAGTTTCTC -3’

ssODN: 5’ -AAGATTCTTCACACACAGGTGAAACCTACTGATGAGAAACTC[TGG]GAGCTGAAGGGCGCCAAGCTAGTGATTGATGTAATTCGTTAT -3’

SMC1A-R586W clone 1 murine embryonic stem cell line sequence at the site of genome editing:

TACATCAATCACTAGCTTGGCCCCCTTCAGCTCCCAGAGTTTCTCATCAGTAGGTTTCACCTGTGTGAAGAATCTTATGAAGCAACAGAATACATAAAGCCAAGGTGAGGGGCCCATAACACTGCCTTGACCCA

SMC1A-R586W clone 2 murine embryonic stem cell line sequence at the site of genome editing:

TACATCAATCACTAGCTTGGCCCCCTTCAGCTCCCAGAGTTTCTCATCAGTAGGTTTCACCTGTGTGAAGAATCTTATGAAGCAACAGAATACATAAAGCCAAGGTGAGGGGCCCATAACACTGCCTTGACCCA

### RNAi

Cells were counted and 5 x 10^5^ were plated per well in 6-well plates. 50nM of SMARTpool siRNA pools (GE Healthcare) targeting *Nipbl* or *Wapl*, 25nM each of SMARTpools targeting *Pds5a* and *Pds5b*, or 50nM of the negative control siGLO reagent, were transfected using DharmaFECT (GE Healthcare) transfection reagent following the manufacturer’s instructions. Cells were collected 48 hours post-transfection to assess knockdown efficiency for all lines and siRNA pools. For proliferation assays, cells transfected with the *Nipbl-* and *Wapl*-targeting siRNA pools were seeded into 6-well plates 24 hours post-transfection, and cells transfected with the *Pds5a-* and *Pds5b*-targeting siRNA pools were seeded 48 hours post-transfection.

### RT-qPCR

To extract RNA, cells were resuspended in 1ml Trizol (Thermo Fisher, 15596018) and incubated for 5 minutes at room temperature and stored at at -80°C, or further processed. 200μl chloroform (Millipore Sigma, St. Louis, MO; C2432) was added and mixed, before centrifugation to separate organic and aqueous phases. The aqueous phase was recovered, mixed with 400μl additional chloroform, and centrifuged. The aqueous phase was recovered and RNA was precipitated by addition of isopropanol. Total RNA was quantified using a NanoDrop instrument (Thermo Fisher). cDNA was prepared with Superscript IV and oligo-d(T) primers (Thermo Fisher, 18091050) according to manufacturer’s instructions. qPCR was performed using SYBRgreen Master Mix on an Applied Biosystems QuantStudio 6 qPCR instrument using primers found in **S5 Table**.

### RNA-seq

RNA was extracted as described above, then cleaned up using the Zymo RNA Clean and Concentrator Kit (Zymo Research, R1013). Libraries were prepared and sequenced by Novogene (Sacramento, CA) on an Illumina NovaSeq 6000 with 150 bp paired end reads.

### Chromatin immunoprecipitation

Cytoplasms of crosslinked cells were lysed by sequential incubations in lysis buffer 1 (50 mM Hepes-KOH pH7.5, 140 mM NaCl, 1mM EDTA, 10% glycerol, 0.5% NP-40, and 0.25% Triton X-100) and lysis buffer 2 (10mM Tris-HCl pH 8, 200mM NaCl, 1mM EDTA, and 0.5 mM EGTA). Nuclei were recovered and washed with shearing buffer (10mM Tris pH 7.5, 1mM EDTA, 0.1% SDS). Nuclei were resuspended in shearing buffer at 30 x 10^6^ cell equivalents/ml, and 1ml of nuclei was sonicated on a Covaris E220 to generate chromatin fragments of 200-1000bp. Following sonication, insoluble material was cleared by high-speed centrifugation.

Prior to addition to chromatin, 10μg antibodies were incubated with 30μL Protein G Dynabeads (Thermo Fisher) for 6–8 hours. Beads were washed three times with 1mL PBS containing 1X complete protease inhibitor cocktail (PIC; Roche, Basel, Switzerland; 11697498001). Chromatin from 1 x 10^7^ cell equivalents was added to antibody conjugated beads, and buffer was adjusted to a total volume of 1ml (ChIP buffer; 15mM Tris pH 7.5, 1.5mM EDTA, 0.1% SDS, 150mM NaCl, 1% Triton X-100, 1x PIC). Chromatin and bead-bound antibodies were rotated together overnight at 4°C.

After overnight incubation, beads were washed with 1 ml ChIP buffer, wash buffer 1 (20mM Tris-HCl pH 8, 500mM NaCl, 2mM, EDTA, 0.1% SDS, and 1% Triton X-100), wash buffer 2 (10mM Tris-HCl pH 8, 250mM LiCl, 1mM EDTA, and 1% NP-40), and wash buffer 3 (10mM Tris pH 8, 1mM EDTA, and 50mM NaCl). For K27me3 ChIPs, the above washes were replaced with 3 washes of 1ml RIPA buffer (50 mM HEPES pH 7.5, 500 mM LiCl, 1 mM EDTA, 1% NP-40 and 0.7% sodium deoxycholate), followed by one wash with wash buffer 3. All wash buffers were supplemented with 1x PIC. Chromatin was eluted from beads by adding elution buffer (50mM Tris pH 8, 10mM EDTA, and 1% SDS) and incubating at 65°C for 1 hour. Crosslinks were reversed through overnight incubation with Proteinase K (Thermo Fisher), after which DNA was purified using a ChIP DNA Clean and Concentrate kit (Zymo, Irvine, CA; D5205). Sequencing libraries were prepared using a Kapa Hyper Prep Kit (Roche) according to manufacturer’s instructions.

### High-throughput sequencing

For ChIP-seq, 50bp single-end reads or 100bp single-end or paired-end sequencing was performed on Illumina (San Diego, CA) Hi-Seq 4000, Hi-Seq 2500, or NovaSeq 6000 platforms using Illumina reagents according to manufacturer’s instructions.

### Co-immunoprecipitation

Co-immunoprecipitation studies were performed using a Nuclear Complex Co-IP Kit (Active Motif, 54001), with modifications, as previously described [68]. Ten μg of each antibody was bound to Protein G Dynabeads (Thermo Fisher, 10009D) for 6–8 hours before application of cellular extracts. Cells were lysed and nuclei permeabilized according to manufacturer’s instructions. Nuclei were then resuspended in nuclear digestion buffer (10mM HEPES pH 7.9, 10mM KCl, 1.5mM MgCl_2_, 340mM Sucrose, and 10% glycerol) and incubated for 15 minutes at 37°C with 10U of benzonase (Millipore Sigma, E1014). Digestion was quenched with addition of EDTA to 5mM, and insoluble components of the nuclear extract were cleared by high-speed centrifugation. Each IP was performed overnight at 4°C, using 250μg of nuclear extract in 500μl of 1x low-stringency IP buffer (Active Motif, 54001). Beads were washed 3x in 1x low-stringency IP buffer supplemented with 100μg/ml BSA, then 3x in 1x low-stringency IP buffer without BSA. Protein was eluted from the beads by incubating for one hour in ChIP elution buffer. Input material was reserved as a control. All buffers following the benzonase treatment were supplemented with 1x PIC. Western blots were performed as described below.

### Subcellular protein fractionation

Subcellular protein fractionation was performed using a Subcellular Protein Fractionation kit (Thermo Fisher, 78840) according to manufacturer’s instructions, with the addition of a wash in 1x PBS between each fractionation step. Fractionated proteins were analyzed by western blotting as described below.

### Western blotting

Nuclear protein extraction and western blotting were performed as previously described [68]. Briefly, cells were collected via scraping and lysed by incubating for 15 minutes in cold Lysis Buffer A (10mM HEPES pH 7.9, 10mM KCl, 0.1mM EDTA, and 0.1mM EGTA, 1x PIC), and intact nuclei were pelleted and recovered via centrifugation. Nuclear protein were extracted by incubating with TEN250/0.1 buffer (50mM Tris-HCl pH 7.5, 250mM NaCl, 5mM EDTA, and 0.1mM NP-40). Insoluble nuclear components were cleared by high-speed centrifugation. For the western blot appearing in **S1 Fig**, a Dounce homogenizer was used to complete cell lysis.

Western blotting was performed as previously described [68]. Protein extracts in 1x Laemmli buffer were loaded into 4–20% pre-cast gradient PAGE gels (Bio-Rad, 4561094) and run to completion in a 25mM Tris, 192mm glycine, 0.1% w/v SDS running buffer (Bio-Rad, 1610732) using a gel electrophoresis apparatus. Protein was transferred onto polyvinylidene fluoride (PVDF) membranes by electrophoresis in 25mM Tris, 192mM glycine, 20% v/v methanol. After transfer, membranes were blocked with 5% milk in Tris-buffered saline (20mM Tris, 150mM sodium chloride) supplemented with 0.1% w/v Tween-20 (TBS-T), and incubated overnight at 4°C with antibodies diluted in the same buffer supplemented with milk. After incubation, membranes were washed with TBS-T and incubated for 1 hour at 4°C with secondary antibody diluted in blocking buffer. After incubation, membranes were washed with TBS-T, then developed using SuperSignal West Pico or Femto (Thermo Fisher) chemiluminescent substrate. Images were acquired using the Amersham Imaging platform (GE Healthcare). After imaging, blots were stripped using Reblot Plus Strong buffer (Millipore Sigma, 2504) according to manufacturer’s instructions before re-probing with additional primary antibodies.

### Hi-C

Hi-C was performed as previously described [68] using the Arima Genomics (San Diego, CA) Hi-C kit. Sequencing was performed on the Illumina NovaSeq platform using either SP or S4 flow cells, with either 50bp or 150bp paired-end reads collected.

## Quantification and statistical analysis

### ChIP-seq analysis and normalization

Reads in.fastq format were aligned to a merged genome containing both mouse genome assembly mm10 and human genome assembly hg38 using bowtie as previously described previously (v 1.2.2) (parameters -v 2 -p 24 -S -m 1 –best–strata) [68,70]. Duplicate sequences were removed using samtools (v 1.9) markdup (-r -s) [71]. A bam file containing only mouse reads was created using samtools view and converted to bed format using bedtools (v 2.26) bamtobed and reads were extended by 200 bp [72]. Extended bed files were used to call peaks using MACS (v 2016-02-15) with a false discovery rate of 1% (macs2 callpeak -f BED -g mm -q 0.01) [73]. For H3K27me3 ChIP-seq, broad peak calling was performed (macs2 broadpeak -f BED -g mm -q 0.01). To obtain a high confidence peak set, called peak summits were expanded by 50 bp on either side using awk and any expanded peak overlapping a repeat element (defined using the Repeat Masker Track from UCSC genome browser) was removed prior to any peak-related analysis. Z-score normalization was performed where indicated using a custom R script [68]. The bed file containing aligned reads was converted to bedgraph using bedtools genomecov (-bga -scale 1/h) before being converted to a bigwig file with bedGraphToBigWig from ucsctools (v 320) [74].

Overlap peak lists were generated by using bedtools intersect on summit files generated by MACS extended by 500 bp on either side for RAD21, K4me3, and K27ac peak sets. H3K27me3 broad peaks were defined as overlapping if they shared ≥1bp identity. Peaks were defined as overlapping a TSS if they fell within 1kb of UCSC-annotated TSSs. Peaks were defined as overlapping a Hi-C loop anchor if the 500bp-extended summit file overlapped with a loop anchor. Average signal plots were generated using deeptools (v 3.0.1) computeMatrix (reference-point for CTCF sites, promoters, and enhancers; scale-regions for meta-loop anchors and insulated domains) followed by deeptools plotProfile [75]. Heatmaps were generated using Deeptools computeMatrix reference-point or scaled-regions followed by Deeptools plotHeatmap. Fingerprint plots were generated using the plotFingerprint routine in Deeptools. Coverage tracks were visualized using the UCSC Genome Browser. Differentially bound sites were identified using DiffBind, as were the correlation coefficients presented in S3A Fig [45]. Locations of insulated neighborhoods (Super-enhancer Domains and Polycomb Domains) were obtained from Dowen et al. (2014), and wildtype CTCF ChIP-seq data were obtained from Justice et al. (2020).

### RNA-seq analysis

RNA-seq was analyzed as previously described [68], with modifications. RNA sequence reads were aligned to mouse genome build mm10 using Star (version 2.6.0a) [76]. Differentially expressed genes were identified using DESeq2 from Bioconductor with an adjusted p-value of 0.10 [77]. Violin plots of differentially expressed genes were generated and analyzed using Prism 8.0 (Graphpad). Heatmaps were generated using the pHeatmap software package in RStudio (RStudio, Inc.). The gProfiler web-based software was used to perform gene ontology analysis using default parameters (Raudvere et al., 2019).

### Hi-C analysis

Initial processing of Hi-C data was performed with the Juicer software package [46]. Reads were aligned using BWA-mem with default parameters, after which PCR duplicates, reads with Q≤30, and self-ligated fragments were filtered out before Hi-C matrices were assembled (S3 Table) [78]. Matrices were visualized using the Juicebox software package [79]. Loops were called using the HiCCUPS algorithm [49] (parameters -p 8,4,2 -i 14,10,6) on Knight-Ruiz balanced matrices at resolutions of 5 kb, 10 kb, and 25 kb, and the resulting list of merged loops was used for subsequent analyses. Contact domains were called using the Arrowhead algorithm [49] with default parameters on Knight-Ruiz balanced matrices at 10 kb resolution. Eigenvectors for analysis and visualization of compartmentalization were calculated by passing aligned reads into the Homer Hi-C analysis software package and using the makeTagDirectory and runHiCpca.pl routines [80,81].

Loop and contact domain positions were compared using the Compare Lists software in the Juicer Tools suite, with a tolerance of 25kb for loop anchor positions and a tolerance of 10kb for contact domain boundaries. Loops called in one genotype were assessed for sub-threshold signal in the other using the HiCCUPsDiff software (parameters -r 5000,10000,25000 -p 8,4,2 -i 14,10,6). Overlaps between loop anchors and other features defined by ChIP-seq were assessed using the bedtools intersect function. Interaction frequency curves were calculated from un-normalized 10kb contact matrices using the Homer analyzeHiC routine (-ifc–res 10000).

### Image analysis

All image analysis was conducted using the Fiji/ImageJ software package [82]. Western blots after coIP experiments were quantified by measuring pixel density for each lane, then normalizing to either wildtype signal for input lanes, or to the signal in the blot for the IP target protein. For westerns run with subnuclear protein fractions, pixel density was measured for each lane, then values for soluble and chromatin-bound were summed as a measure of total nuclear protein. Chromatin-bound pixel density was then represented as a fraction of the sum of soluble and chromatin-bound signal. For westerns after both coIP and fractionation experiments, background was calculated for each lane by making two measurements of non-specific signal within a lane. These measurements were averaged and subtracted from the signal for the specific band of interest. EB area was determined by using the Fiji area and Feret diameter measurement functions.

### Statistical analysis

Unless otherwise noted, statistics were performed using Prism 8 software (Graphpad, San Diego, CA) or R version 3.4 (The R Project) running within RStudio version 1.2 (RStudio, Inc., Boston, MA).

## Supporting information

S1 Fig Related to Fig 1. **Phenotypic changes in SMC1A-R586W mESCs. A** Position of R586 within the cohesin complex (PDB:2WD5)[13,34]. **B** Alignment of the R586 region of SMC1A in various eukaryotes. **C** Expression of *Smc1a* transcripts in R586W mESCs. n = 3 biological replicates. **D** Expression of core Cohesin subunits in SMC1A-R586W mESCs **E** Principal component analysis of RNA-seq data in wildtype and R586W ESCs. **F** RT-qPCR analysis of gene expression changes for pluripotency genes in R586W cl1 and cl2. *, p<0.05 as measured by two-way ANOVA. n = 3 biological replicates. **G** Expression of Super-enhancer domain (SD) and Polycomb Domain (PD) genes in RNA-seq data. ****, p<0.0001 as measured by Kruskal-Wallis test. **H** Embryoid body size after 3 days of differentiation in hanging-droplet cultures in the absence of LIF. ****, p<0.0001 as measured by Kruskal-Wallis test. Data merged from three biological replicates, with total number of measurements given. **I** Proliferation rate during 72 hours as measured by hemacytometer. p<0.05 at 72 hours between wildtype and R586W clone 2 mESCs as measured by two-way ANOVA. n = 5 biological replicates. **J** DNA content as measured by propidium iodide staining in the indicated lines. P values >0.05 for all comparisons between genotypes as measured by 2-way ANOVA. **K** Cardiomyocyte differentiation in WT and SMC1A-R586W mESCs. Error bars ± 1 s.d. Differences n.s. as measured by two-way ANOVA. n = 2 biological replicates for each group. Numerical data are presented in **S6 Table**.
(TIF)

S2 Fig Related to Fig 2. **Histone modifications in SMC1A-R586W mESCs. A** UCSC Genome Browser coverage tracks showing K27me3 distribution at the *Nes* locus in mESCs. **A** Overlap of K4me3 peaks called in wildtype and SMC1A-R586W mESCs **B** Overlap of K4me3 peaks, **C** K27ac peaks, and **D** K27me3 peaks in wildtype and SMC1A-R586W mESCs. **E** Differential binding analysis performed with Diffbind [45] for K4me3, **F** K27ac, and **G** K27me3. Red dots indicate p<0.05. **H** Expression of DEGs with TSSs containing a called K4me3 peak only in WT, only in SMC1A-R586W mESCs, or in both. **, p<0.01 and ****, p<0.0001 as measured by Kruskal-Wallis test. **I** Expression of DEGs with TSS containing a K27ac peak only in WT, only in SMC1A-R586W mESCs, or in both (with increased signal in SMC1A-R586W mESCs as measured by Diffbind ‘Inc. in R586W’). ****, p<0.0001 as measured by Kruskal-Wallis test. **J** Expression of DEGs with TSSs containing a called K27me3 peak only in WT, only in SMC1A-R586W mESCs, or in both. Differences n.s. as measured by Kruskal-Wallis test.
(TIF)

S3 Fig Related to Fig 3. **RAD21 ChIP-seq in SMC1A-R586W mESCs. A** Correlation analysis and **B** principal component analysis of wildtype and R586W individual RAD21 ChIP-seq replicates. **C** Fingerprint analysis of individual and **D** merged RAD21 ChIP-seq replicates in wildtype and R586W mESCs. **E** Heatmaps displaying RAD21 signal in individual replicates across same set of peaks as in **Fig 3B**. **F** K27ac signal at K27ac peaks ≤1kb from a TSS. **G** K27ac signal at K27ac peaks >1kb from a TSS. **H** Overlap of indicated histone modification with WT RAD21 peaks or a merged list of SMC1A-R586W RAD21 peaks. **I** RAD21 ChIP signal at TSSs associated with upregulated or **J** downregulated genes in wildtype and R586W mESC clones.
(TIF)

S4 Fig Related to Fig 4. **Three-dimensional genome organization in SMC1A-R586W mESCs. A** Hi-C heatmaps at 10kb resolution centered around the *HoxA* locus for individual replicates in wildtype and **B** SMC1A-R586W mESCs. Cyan dots indicate positions of called loops in merged data. **C** Cumulative distribution plot of contact domain size in wildtype and SMC1A-R586W mESCs. **D** Insulation scores and **E** directionality indices at contact domain boundaries in merged wildtype and SMC1A-R586W data sets. **F** Cumulative distribution plot of DNA loop size in wildtype and SMC1A-R586W mESCs. **G** APA scores in WT and SMC1A-R586W mESCs using WT-called DNA loops in specified size ranges. **H** Overlap of DNA loop anchors with histone modifications and **I** CTCF and/or RAD21 in wildtype and SMC1A-R586W mESCs. **J** Compartment analysis along chromosome 1 and **K** genome-wide in wildtype and SMC1A-R586W mESCs.
(TIF)

S5 Fig Related to Figs 2, 3 and 4. **Changes in genome organization and cohesin enrichment at AML tumor suppressor loci. A** Difference map (R586W –WT) of Hi-C signal at the *Dmnt3a* locus. Data presented at 10kb resolution. **B** Contact domain calls, RAD21 and K27ac ChIP-seq signal at the *Dnmt3a* locus in WT and SMC1A-R586W mESCs. **C** Difference map (R586W –WT) of Hi-C signal at the *Tet2* locus. Data presented at 10kb resolution. **D** Contact domain calls, RAD21 and K27ac ChIP-seq signal at the *Tet2* locus in WT and SMC1A-R586W mESCs.
(TIF)

S6 Fig Related to Fig 5. **Physical and functional interactions between cohesin and cohesin accessory proteins in SMC1A-R586W mESCs. A-E** Quantification of 3 biological replicates of the coIP experiment presented in **Fig 5A**. Graphs in **A-C** provide quantification of the indicated bands represented by the blot on the left in **Fig 5A**. Graphs on **D-E** provide quantification of the indicated bands represented in the blot on the right in **Fig 5A**. All differences between wildtype and SMC1A-R586W are not significant as measured by two-way ANOVA. **F** Non-transformed cell count data from experiment in **Fig 5G**. **G** Ratio of si*Wapl* to siControl proliferation in wildtype and SMC1A-R586W mESCs. Differences n.s. as measured by two-way ANOVA. **H** *Wapl* knockdown relative to siControl-treated cells as measured by RT-qPCR in si*Nipbl* and si*Wapl* mESCs. n = 2 biological replicates for all except R586W clone 2, where n = 1 biological replicate. **I** Western blots were performed with the indicated antibodies on material recovered from the indicated IP experiments. Data are representative of two biological replicates. **J** Non-transformed cell count data from experiment in **5J**. Numerical data are presented in **S6 Table**.
(TIF)

S1 Table Differentially expressed genes in SMC1A-R586W cl1 and cl2. (XLSX)

S2 Table QC information for RNA-seq and ChIP-seq data generated in this study. (XLSX)

S3 Table Gene ontology analysis of genes differentially expressed in both SMC1A-R586W clones. (XLSX)

S4 Table QC data on Hi-C libraries prepared from wildtype and SMC1A-R586W mESCs. (XLSX)

S5 Table Oligonucleotides used for RT-qPCR in this study. (XLSX)

S6 Table Source data. (XLSX)
