# Supplementary figures and images for: A cohesin cancer mutation reveals a role for the hinge domain in genome organization and gene expression
Source: PLoS Genet. 2021 Mar 24;17(3):e1009435. doi: 10.1371/journal.pgen.1009435 (PMC7990204; doi:10.1371/journal.pgen.1009435)

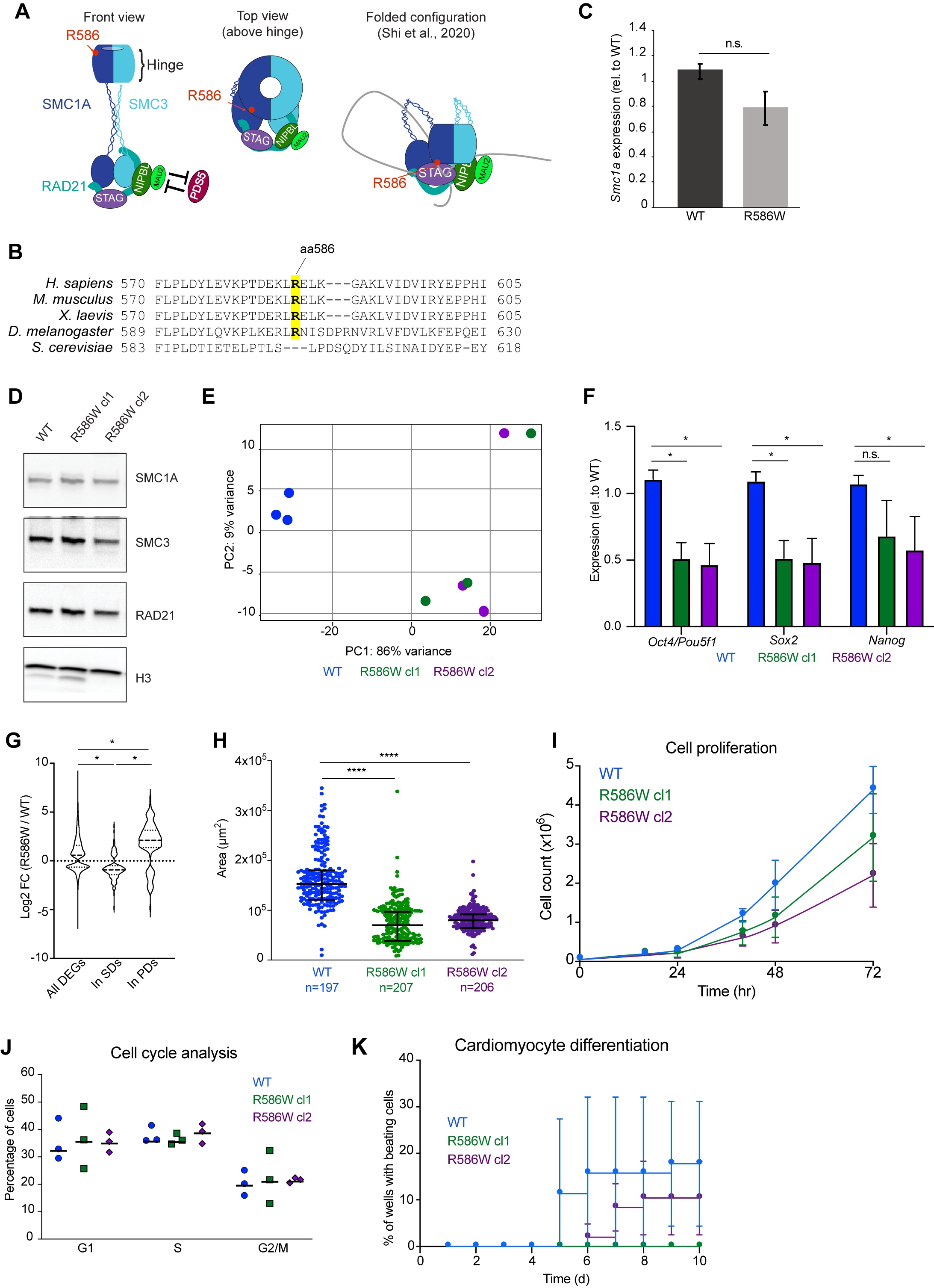

Supplement: S1 Fig — Phenotypic changes in SMC1A-R586W mESCs. A Position of R586 within the cohesin complex (PDB:2WD5)[13,34]. B Alignment of the R586 region of SMC1A in various eukaryotes. C Expression of Smc1a transcripts in R586W mESCs. n = 3 biological replicates. D Expression of core Cohesin subunits in SMC1A-R586W mESCs E Principal component analysis of RNA-seq data in wildtype and R586W ESCs. F RT-qPCR analysis of gene expression changes for pluripotency genes in R586W cl1 and cl2. *, p<0.05 as measured by two-way ANOVA. n = 3 biological replicates. G Expression of Super-enhancer domain (SD) and Polycomb Domain (PD) genes in RNA-seq data. ****, p<0.0001 as measured by Kruskal-Wallis test. H Embryoid body size after 3 days of differentiation in hanging-droplet cultures in the absence of LIF. ****, p<0.0001 as measured by Kruskal-Wallis test. Data merged from three biological replicates, with total number of measurements given. I Proliferation rate during 72 hours as measured by hemacytometer. p<0.05 at 72 hours between wildtype and R586W clone 2 mESCs as measured by two-way ANOVA. n = 5 biological replicates. J DNA content as measured by propidium iodide staining in the indicated lines. P values >0.05 for all comparisons between genotypes as measured by 2-way ANOVA. K Cardiomyocyte differentiation in WT and SMC1A-R586W mESCs. Error bars ± 1 s.d. Differences n.s. as measured by two-way ANOVA. n = 2 biological replicates for each group. Numerical data are presented in S6 Table. (TIF) [file pgen.1009435.s001.tif]

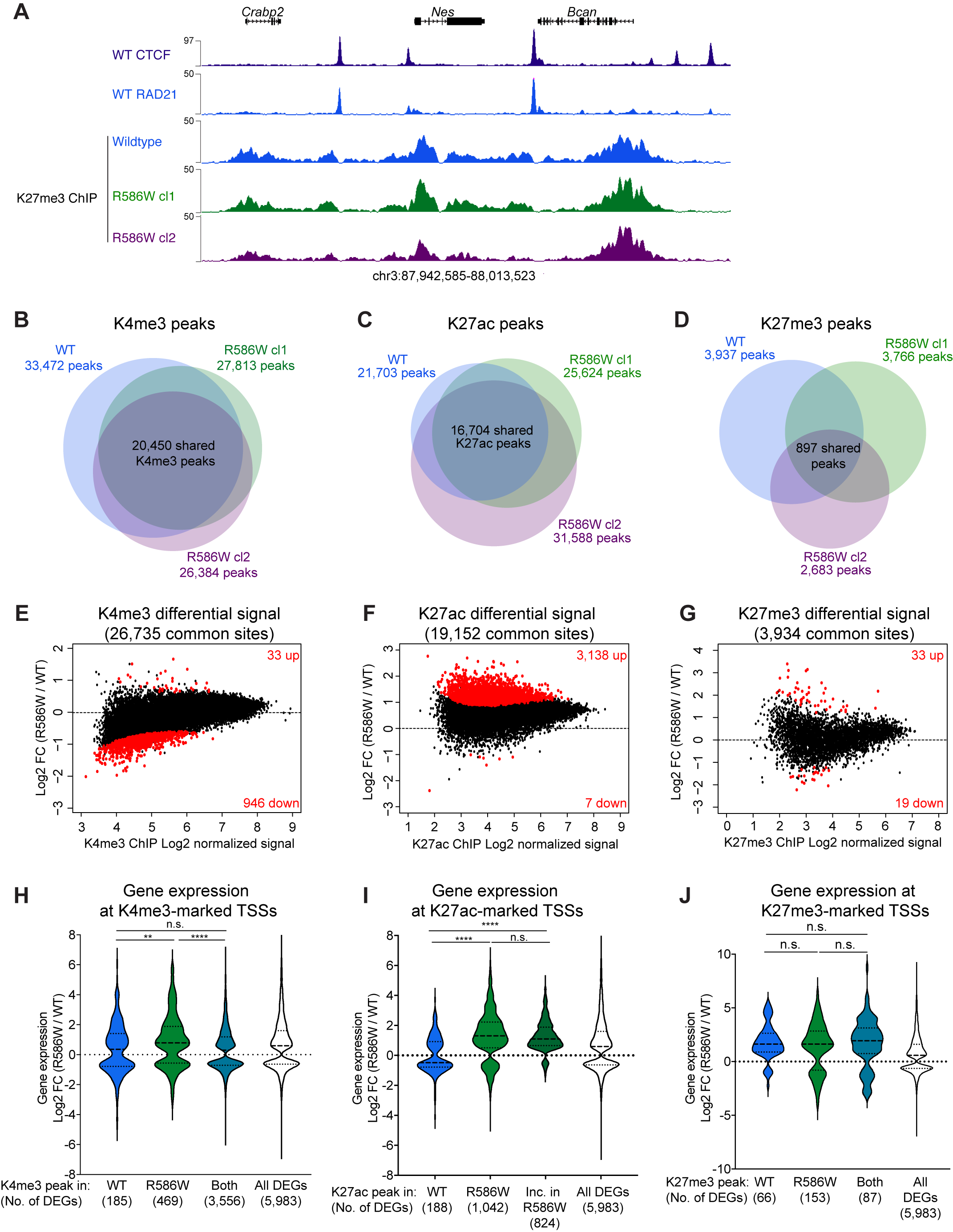

Supplement: S2 Fig — Histone modifications in SMC1A-R586W mESCs. A UCSC Genome Browser coverage tracks showing K27me3 distribution at the Nes locus in mESCs. A Overlap of K4me3 peaks called in wildtype and SMC1A-R586W mESCs B Overlap of K4me3 peaks, C K27ac peaks, and D K27me3 peaks in wildtype and SMC1A-R586W mESCs. E Differential binding analysis performed with Diffbind [45] for K4me3, F K27ac, and G K27me3. Red dots indicate p<0.05. H Expression of DEGs with TSSs containing a called K4me3 peak only in WT, only in SMC1A-R586W mESCs, or in both. **, p<0.01 and ****, p<0.0001 as measured by Kruskal-Wallis test. I Expression of DEGs with TSS containing a K27ac peak only in WT, only in SMC1A-R586W mESCs, or in both (with increased signal in SMC1A-R586W mESCs as measured by Diffbind ‘Inc. in R586W’). ****, p<0.0001 as measured by Kruskal-Wallis test. J Expression of DEGs with TSSs containing a called K27me3 peak only in WT, only in SMC1A-R586W mESCs, or in both. Differences n.s. as measured by Kruskal-Wallis test. (TIF) [file pgen.1009435.s002.tif]

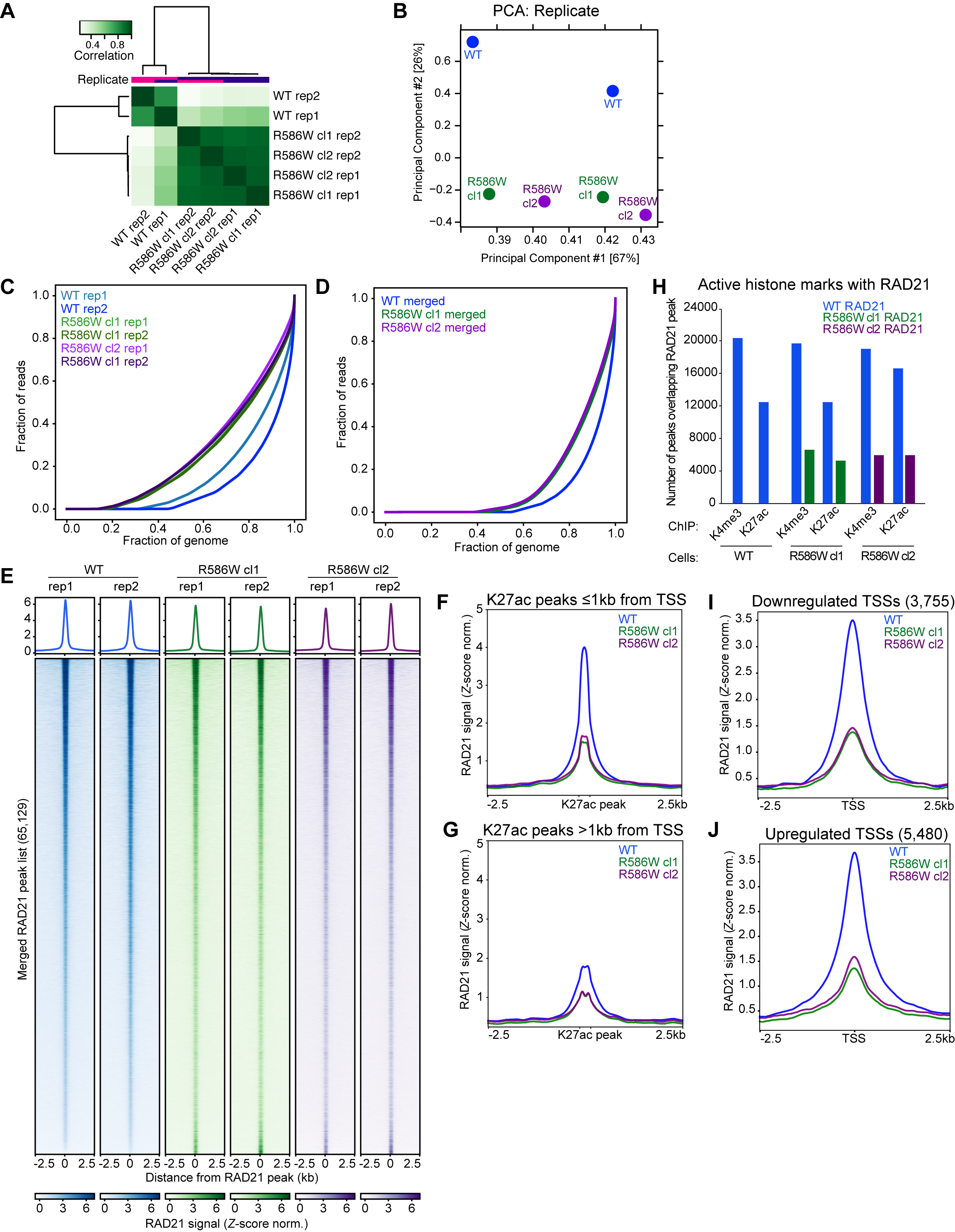

Supplement: S3 Fig — RAD21 ChIP-seq in SMC1A-R586W mESCs. A Correlation analysis and B principal component analysis of wildtype and R586W individual RAD21 ChIP-seq replicates. C Fingerprint analysis of individual and D merged RAD21 ChIP-seq replicates in wildtype and R586W mESCs. E Heatmaps displaying RAD21 signal in individual replicates across same set of peaks as in Fig 3B. F K27ac signal at K27ac peaks ≤1kb from a TSS. G K27ac signal at K27ac peaks >1kb from a TSS. H Overlap of indicated histone modification with WT RAD21 peaks or a merged list of SMC1A-R586W RAD21 peaks. I RAD21 ChIP signal at TSSs associated with upregulated or J downregulated genes in wildtype and R586W mESC clones. (TIF) [file pgen.1009435.s003.tif]

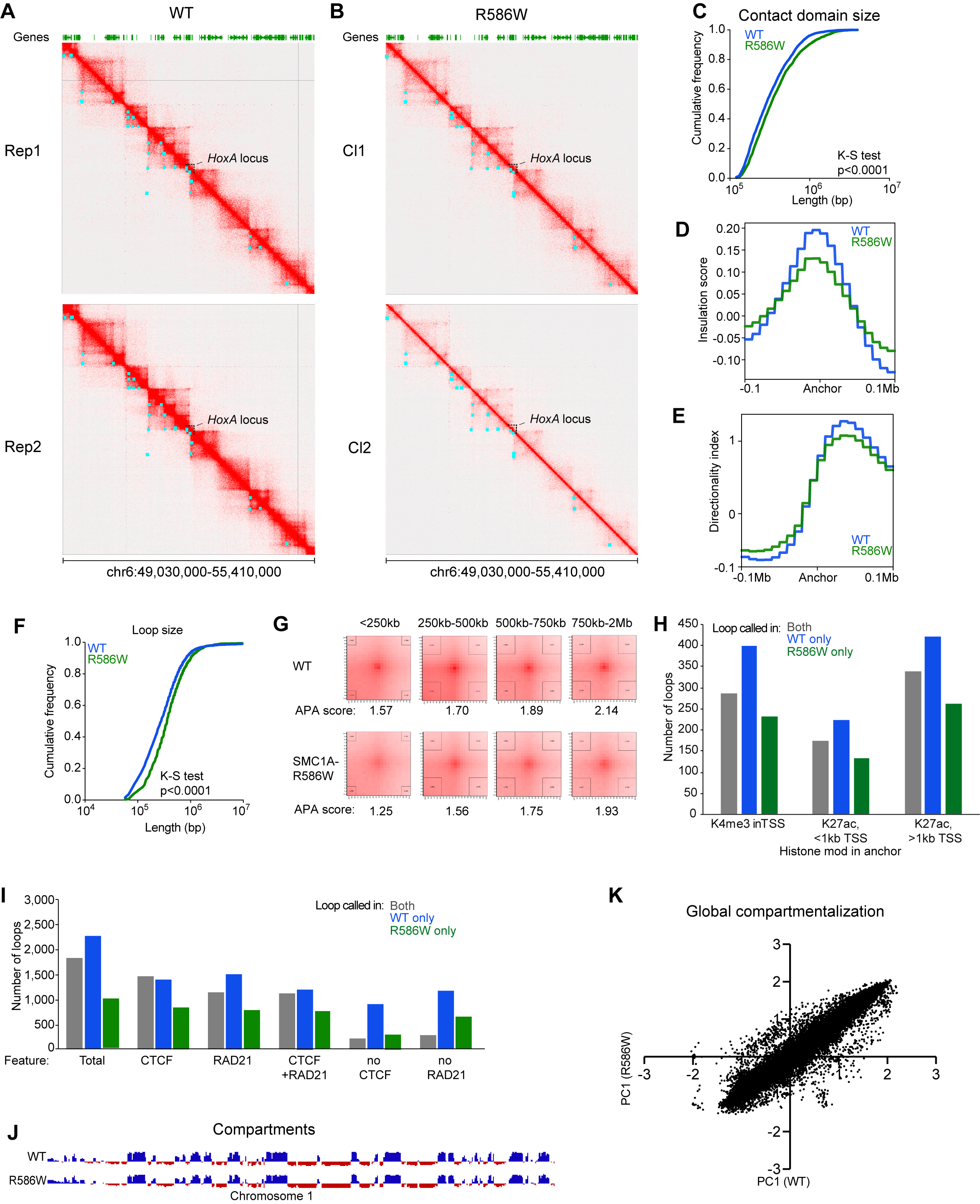

Supplement: S4 Fig — Three-dimensional genome organization in SMC1A-R586W mESCs. A Hi-C heatmaps at 10kb resolution centered around the HoxA locus for individual replicates in wildtype and B SMC1A-R586W mESCs. Cyan dots indicate positions of called loops in merged data. C Cumulative distribution plot of contact domain size in wildtype and SMC1A-R586W mESCs. D Insulation scores and E directionality indices at contact domain boundaries in merged wildtype and SMC1A-R586W data sets. F Cumulative distribution plot of DNA loop size in wildtype and SMC1A-R586W mESCs. G APA scores in WT and SMC1A-R586W mESCs using WT-called DNA loops in specified size ranges. H Overlap of DNA loop anchors with histone modifications and I CTCF and/or RAD21 in wildtype and SMC1A-R586W mESCs. J Compartment analysis along chromosome 1 and K genome-wide in wildtype and SMC1A-R586W mESCs. (TIF) [file pgen.1009435.s004.tif]

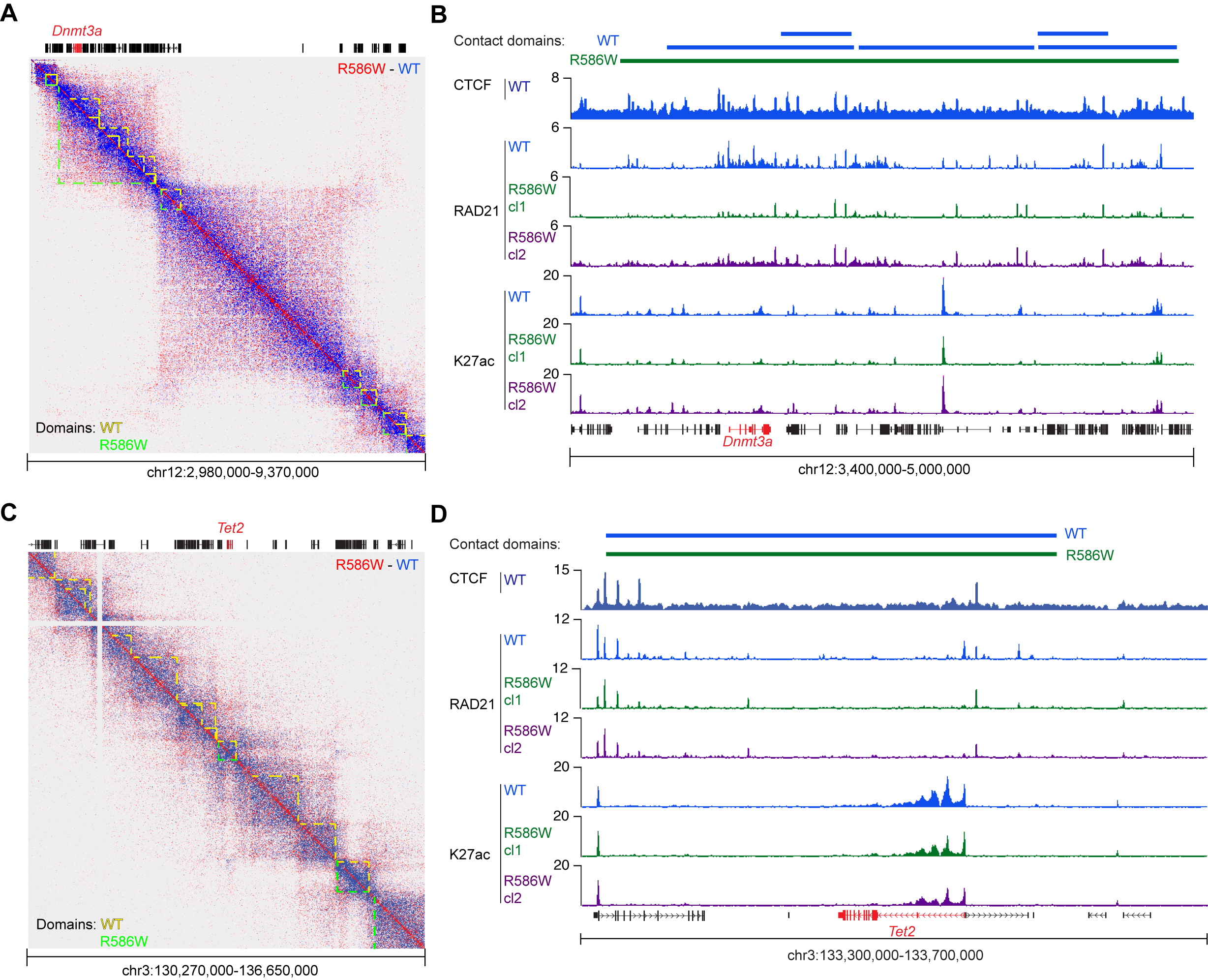

Supplement: S5 Fig — Changes in genome organization and cohesin enrichment at AML tumor suppressor loci. A Difference map (R586W –WT) of Hi-C signal at the Dmnt3a locus. Data presented at 10kb resolution. B Contact domain calls, RAD21 and K27ac ChIP-seq signal at the Dnmt3a locus in WT and SMC1A-R586W mESCs. C Difference map (R586W –WT) of Hi-C signal at the Tet2 locus. Data presented at 10kb resolution. D Contact domain calls, RAD21 and K27ac ChIP-seq signal at the Tet2 locus in WT and SMC1A-R586W mESCs. (TIF) [file pgen.1009435.s005.tif]

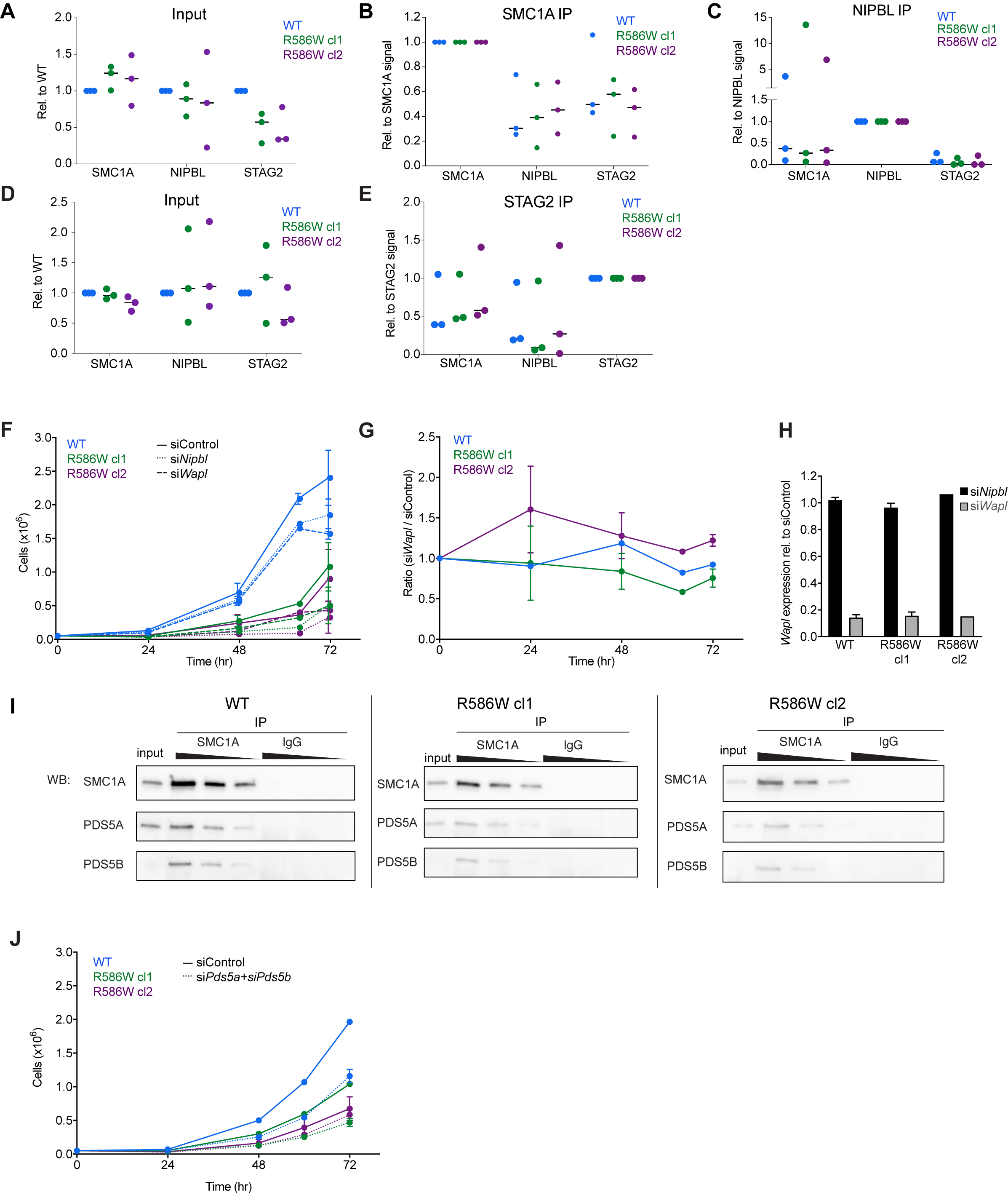

Supplement: S6 Fig — Physical and functional interactions between cohesin and cohesin accessory proteins in SMC1A-R586W mESCs. A-E Quantification of 3 biological replicates of the coIP experiment presented in Fig 5A. Graphs in A-C provide quantification of the indicated bands represented by the blot on the left in Fig 5A. Graphs on D-E provide quantification of the indicated bands represented in the blot on the right in Fig 5A. All differences between wildtype and SMC1A-R586W are not significant as measured by two-way ANOVA. F Non-transformed cell count data from experiment in Fig 5G. G Ratio of siWapl to siControl proliferation in wildtype and SMC1A-R586W mESCs. Differences n.s. as measured by two-way ANOVA. H Wapl knockdown relative to siControl-treated cells as measured by RT-qPCR in siNipbl and siWapl mESCs. n = 2 biological replicates for all except R586W clone 2, where n = 1 biological replicate. I Western blots were performed with the indicated antibodies on material recovered from the indicated IP experiments. Data are representative of two biological replicates. J Non-transformed cell count data from experiment in 5J. Numerical data are presented in S6 Table. (TIF) [file pgen.1009435.s006.tif]
